# Machine learning for fault analysis in rotating machinery: A comprehensive review

**DOI:** 10.1016/j.heliyon.2023.e17584

**Published:** 2023-06-22

**Authors:** Oguzhan Das, Duygu Bagci Das, Derya Birant

**Affiliations:** a National Defence University, Air NCO Higher Vocational School, Department of Aeronautics Sciences, Izmir, Turkey; b Ege University, Ege Vocational School, Department of Computer Programming, Izmir, Turkey; c Dokuz Eylül University, Department of Computer Engineering, Izmir, Turkey

**Keywords:** Intelligent fault diagnosis, Rotating machine, Transfer learning, Machine learning, Deep learning, Challenges and future directions

## Abstract

As the concept of Industry 4.0 is introduced, artificial intelligence-based fault analysis is attracted the corresponding community to develop effective intelligent fault diagnosis and prognosis (IFDP) models for rotating machinery. Hence, various challenges arise regarding model assessment, suitability for real-world applications, fault-specific model development, compound fault existence, domain adaptability, data source, data acquisition, data fusion, algorithm selection, and optimization. It is essential to resolve those challenges for each component of the rotating machinery since each issue of each part has a unique impact on the vital indicators of a machine. Based on these major obstacles, this study proposes a comprehensive review regarding IFDP procedures of rotating machinery by minding all the challenges given above for the first time. In this study, the developed IFDP approaches are reviewed regarding the pursued fault analysis strategies, considered data sources, data types, data fusion techniques, machine learning techniques within the frame of the fault type, and compound faults that occurred in components such as bearings, gear, rotor, stator, shaft, and other parts. The challenges and future directions are presented from the perspective of recent literature and the necessities concerning the IFDP of rotating machinery.

## 1 Introduction

The concept of Machine Learning (ML) is an essential branch of artificial intelligence that helps to solve numerous problems in various disciplines including engineering, health, finance, education, and military in which optimization, prediction, and assessment are required. ML techniques can be categorized into supervised, unsupervised, semi-supervised, and reinforcement learning. In general, supervised learning addresses regression and classification procedures while unsupervised learning is typically employed in association and cluster problems. Various ML techniques are practiced for different kinds of problems. The most commonly used algorithms are Deep Neural Networks (DNN) algorithms [[1], [2], [3], [4], [5]], Artificial Neural Networks (ANN) [[6], [7], [8]], AdaBoost (AB) [8,9], Random Forest (RF) [10,11], Support Vector Machines (SVM) [[12], [13], [14]], K Nearest Neighbors (KNN) [12,13,15], and Decision Trees (DT) [16,17]. As the concept of the Fourth Industrial Revolution or Industry 4.0 is introduced, the use of ML becomes even more critical, especially for the industry itself. Industry 4.0 aims to constitute smart factories where all industrial procedures are conducted by establishing communication among industrial systems and humans. Apart from the traditional techniques, the communication procedure is conducted by the cooperation between humans and the machines or the elements of the machines through the internet [18]. Such communication is provided by software and networked sensors to come up with a better business. The adaptation and adoption of Industry 4.0 in the industrial divisions (e.g. manufacturing, maintenance, quality assurance, logistics, finance, human resources, etc.) require developing intelligent models that should operate productively and cost-effectively. ML helps to satisfy these requirements by bringing powerful and effective techniques that can solve complex problems.

In the field of Industry 4.0-based maintenance, the concept of the Industrial Internet of Things (IIoT) and Cyber-Physical Systems play a major role. These technologies associate data and machine learning to build intelligent techniques for condition monitoring of the machines. Hence, developing IFDP methods elicits an opportunity to predict, plan, and apply the maintenance procedure as effectively as possible. On the other hand, there are several challenges that have to be overcome. These challenges can be split into organizational challenges, architectural challenges, infrastructural challenges, content and contextual challenges, and integration challenges [19]. Machine learning-related challenges can be summed up into architectural and content and contextual challenges. As it is anticipated, the foremost challenge is to constitute an implementable effective IFDP model [20]. The selection, tuning, or modification of an IFDP technique is compelling in various ways. It is essential to assess the IFDP method based on its success, time consumption, explainability, applicability, and generalizability. Hence, it is first beneficial to understand the fundamentals of a chosen technique including its mathematical background, applicability to a given problem, tunable parameters, pros, and cons. In addition, it is critical to pick or constitute an intelligent model that can be operated without requiring a heavy computational load that may result in an increase in costs due to the necessity of high-end hardware. During selecting or developing a model, it is also needed to check its compatibility with the data used for monitoring. Some visual or signal data need to be processed to be employable as input to IFDP models. In addition, a selected IFDP approach may give fallacious results when small data is used. For instance, deep learning approaches generally give better results when they are fed with bigger amounts of data. An explainable (XAI) IFDP model would be favorable since it provides users with an in-depth understanding related to the way of learning of the IFDP model. Following the selection of IFDP, it has to be tuned or modified if necessary to achieve a robust model which successfully analyzes the machine in a short time. At this step, it is also necessary to pay attention to overfitting where an intelligent method gives accurate results by memorizing instead of learning. The challenges do not come to an end when an effective IFDP is obtained. The proposed approach has to be tested in real-world settings if it is not assessed yet. Based on such challenges, this paper provides a comprehensive overview of ML-based fault diagnosis and the prognosis of rotating machines in industries to present the recent situation related to this field and address the challenges, shortcomings, and future directions.

Rotating machines have an essential role to accomplish various purposes in the industry. These machines are the backbone of mechanized and autonomous production that provides fast and easy-to-reach all products that humanity needs. A sudden outage of the rotating machines due to a fault or a failure not only disrupt the supply chain but also causes enormous amounts of costs. Therefore, it is essential to determine a maintenance strategy and arrange a schedule meticulously. For this reason, industries adopted many different maintenance strategies. As the IIoT is introduced, these maintenance strategies evolved and become even more significant. Especially the impact of digital technologies on Predictive Maintenance (PdM) improved its preferability significantly. Consequently, developing the IIoT integrated and ML-based PdM models attracted researchers, and therefore, various studies are published in which different intelligent fault detection, isolation, diagnosis, and prognosis techniques are proposed, examined, and interpreted.

An intelligent model should be effective, accurate, fast, cheap, low-computational cost, and real-world applicable for fault detection, fault isolation, fault diagnosis, and fault prognosis of rotating machines. To develop Intelligent Fault Diagnosis and Prognosis (IFDP) model, researchers need to consider suitable data sources, preprocessing techniques, machine learning algorithms, and optimization algorithms. For this purpose, they deemed vibration [11,14,15,21,22], acoustic [11,23,24], thermal [13], current [6,7,9,25,26], pressure [27], and other characteristic data [[27], [28], [29]] as the main source for IFDP of rotating machines. Afterwards, the distinctive features are extracted by employing feature extraction methods such as statistical feature extraction [[30], [31], [32]], Fourier Transform [[33], [34], [35]], Wavelet Transform [[36], [37], [38]], Empirical Mode Decomposition [28,39,40] or other techniques [6,7,41,42]. The features may also be extracted automatically by employing deep learning approaches including convolutional neural networks, autoencoders, long-short term machines, etc. [[43], [44], [45]] Following the feature extraction, a suitable machine learning approach is necessary to be considered. Researchers employed and improved numerous machine learning approaches [[46], [47], [48]] including deep learning [[49], [50], [51]] and ensemble learning [12,14,52] methods.

The literature comprises several review studies in which the IFDP approaches regarding rotating machines are discussed. Most of these studies focused on various aspects including intelligent approaches [[53], [54], [55], [56], [57], [58]] and data preparation/processing [54,55,[58], [59], [60]] regarding the past, present, and future timeline [58]. On the other hand, few reviews considered fault-specificity [53,57], the component of the rotating machinery [54,58,59,61], performance metrics [[53], [54], [55], [56]], and fault analysis strategies [62]. However, a gap still exists regarding these concepts because most of those works focused on a specific component or a group of faults related to a single component [57,61]. Besides, the relationship and the importance among the fault specificity, selected intelligent approach, data sources, data processing, data fusion, performance metrics, and fault analysis strategy is not discussed yet. Another significant issue is the compound faults that involve multi-fault occurrences that take place at the same time in the same or different components. Hence, this study differs from other reviews by addressing the recent IFDP approaches related to rotating machines and proposing the challenges and future directions within the concept of fault analysis including fault diagnosis (fault detection, localization, and identification) and fault prognosis (fault severity assessment and remaining useful life) regarding machine learning approaches, rotating machine components, single and compound fault-specificity, data sources, and fusion techniques. The contributions of this review are summarized as follows.

(i) Presenting a recent and comprehensive discussion regarding IFDP in bearing, gear, rotor, stator, shaft, and other rotating machine components.
(ii) Discussing the studies concerning various performance metrics and fault specificity regarding intelligent fault diagnosis for the first time.
(iii) Investigating the approaches and performance metrics regarding intelligent fault prognosis of rotating machines for the first time.
(iv) Examining the literature considering the pursued fault analysis strategies for IFDP of rotating machines including specific faults and compound faults for the first time.
(v) Reviewing the literature regarding the relationship between the developed intelligent models and compound faults for the first time.
(vi) Addressing the challenges and the future directions related to IFDP of rotating machines.

This review discusses the following research questions (RQ):

RQ1 Which rotating machine components are mostly considered in IFDP with ML?

RQ2 What kind of faults are detected, located, identified, and subjected to prognosis with ML?

RQ3 What are the most common data sources used in the field of IFDP of rotating machinery?

RQ4 What fusion techniques are considered for different data sources in the IFDP of rotating machinery?

RQ5 What feature extraction techniques are adopted for the IFDP of rotating machinery?

RQ6 What are the most common machine learning approaches used in the field of IFDP of rotating machinery?

RQ7 What are the most common machine learning techniques used in the field of IFDP of rotating machinery?

RQ8 Which approach and performance metrics are commonly considered to assess ML approaches built for the IFDP of rotating machinery?

RQ9 What are the most suitable maintenance strategies for IFDP of rotating machinery?

RQ10 What are the main challenges in the field of IFDP of rotating machinery?

RQ11 What are the possible research directions for IFDP of rotating machinery?

The rest of the paper is organized as follows. Section 2 presents the considered research methodology including the selection procedure of the reviewed studies, publication trends over time, and an overview of the process of the rotating machinery fault analysis. The number of the most relevant studies published in the last decade is presented. Section 3 introduces the maintenance strategies including their conceptual definitions and their suitability for intelligent fault diagnosis and prognosis procedures. Section 4 proposed fault analysis strategies by explaining the differences among fault detection, fault isolation, fault diagnosis, and fault prognosis. Besides, the techniques regarding each fault analysis strategy are briefly explained. Section 5 comprises the component-wise IFDP procedures of rotating machines where the IFDP studies are reviewed based on the considered fault type and component. In addition, the complexity of the faults for each component in terms of frequency spectrums. Section 6 presents the IFDP of rotating machine literature from the perspective of data sources, data fusion, feature extraction techniques, and machine learning concepts. Section 7 summarizes the study and points out the challenges that existed in the field of IFDP of rotating machines. Finally, Section 8 presents the research directions in a wide perspective for future works related to this field.

## 2 Research methodology

### 2.1 Systematic literature review methodology

**Fig. 1:**
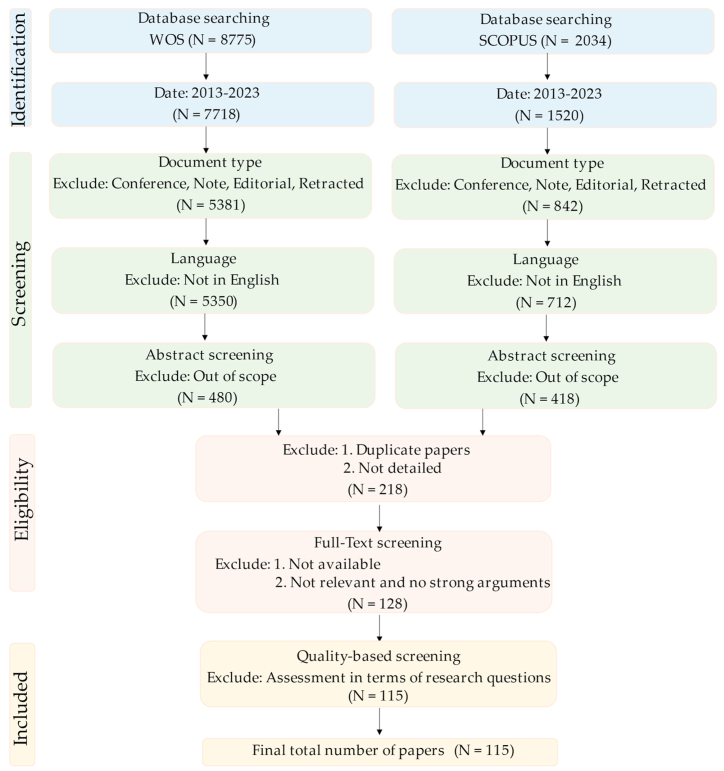
Systematic literature review process.

Fig. 1 shows the PRISMA (Preferred Reporting Items for Systematic Reviews and Meta-Analyses) flow diagram that illustrates the identification, screening, eligibility, and inclusion of studies. The initial search on Web of Science (WOS) and SCOPUS databases returned a large number of papers, totaling 8775 and 2034, respectively. We selected these databases since they are widely regarded as the standard and greatest authority for scientific research.

Thereafter, the retrieved documents were filtered by years (2013–2023) to focus on state-of-the-art studies. After excluding conference papers, notes, editorial materials, meeting abstracts, and retracted papers, a total of 5381 and 842 peer-reviewed journal articles and book chapters were obtained for the review since they usually present a greater level of detail. Following this, a small minority of papers were specifically excluded since they were not in English. After the abstract screening, approximately 1000 studies were deemed eligible for the scope of this review. The papers that do not present sufficient information about the study and duplicates were excluded resulting in 218 articles. The rest of the papers were scanned for relevancy and checked whether they will contribute valuable knowledge to this review. Furthermore, studies for which full texts were not available were excluded. This resulted in 128 research papers suitable for full-text reading. Upon the full-text reading, 18 papers seemed ineligible due to the lack of considerable scientific contribution and detailed information about the research questions, and the rest of the 115 papers were included to be used in the assessment of the review questions.

### 2.2 Publication volumes

**Fig. 2:**
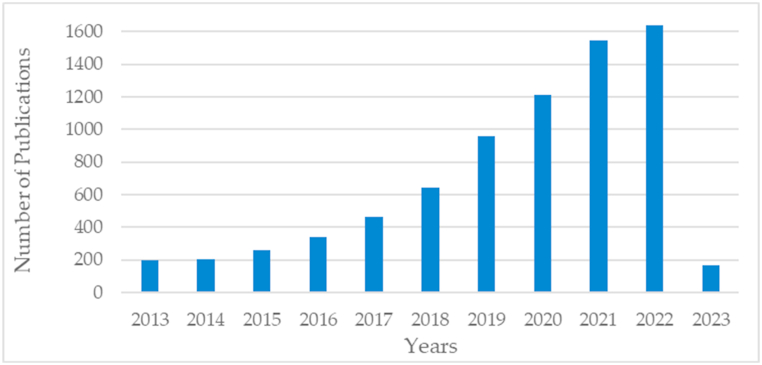
The publication trend in the field over time.

Fig. 2 shows the publication trend in the field of fault analysis over the last decade in terms of the cumulative number of papers published. As seen in Fig. 2, the number of studies geometrically increases, and that reflects the popularity of fault diagnosis and prognosis of rotating machines. For example, while approximately 200 relevant papers were published in 2014, that number was almost 1640 in 2022, which is a significant increase. When analyzing the proportion of publications for each year, continuous growth can be observed from 2020 to 2022, with high proportions of 15.9%, 20.24%, and 21.45%, respectively. Thus, fault analysis has recently received increasing attention worldwide due to its extensive applications and has become a field of research increasingly hot and popular. In the forthcoming years, machine learning will become even more significant due to the presence of a huge volume of fault analysis-related data.

### 2.3 The process of rotating machinery fault analysis

**Fig. 3:**
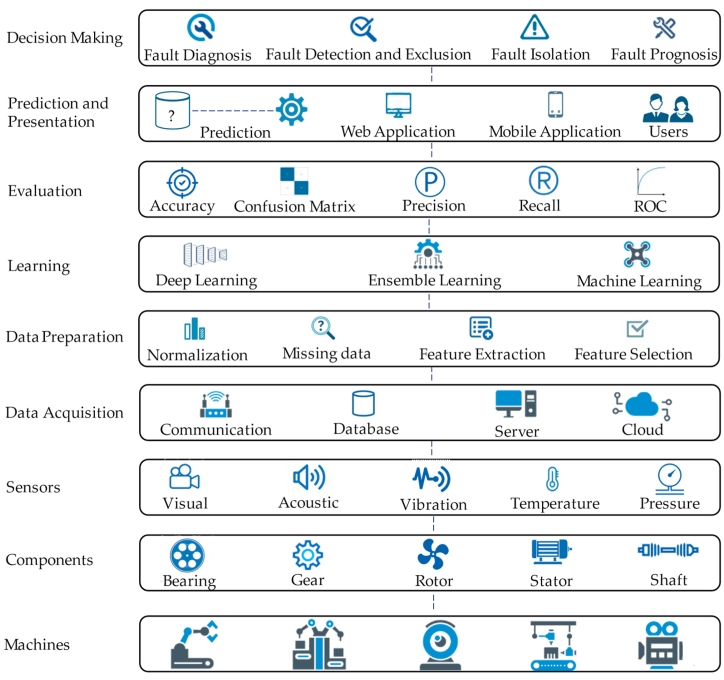
The overall process for rotating machinery fault analysis.

As shown in Fig. 3, the process of rotating machinery fault analysis consists of the sequence of individual parts, including data acquisition, data preparation, learning from data, evaluation, prediction, presentation of the results, and decision-making. The data acquisition phase highly depends on the machinery component, the type of sensor, and the way the data is transferred and stored. Common mechanical components that have been considered in fault diagnosis systems include bearing, gear, shaft, belt-pulley, and induction motors, especially rotor and stator. The type of sensor is selected with an awareness of machine failure modes and the related warning indicators. Typical warning signals in rotating machinery are usually handled with acoustic, vibration, temperature, and pressure sensors, as well as visual devices. Collected data is transferred via a communication technology (i.e., Wi-Fi, Bluetooth) to the server and stored in a database in the cloud environment. Signal records are a collection of time-indexed data instances acquired across a time period. Since this data contains raw values, its current format does not directly give the essence of the data and does not provide meaningful information about the faults. Therefore, signal processing is usually required to prepare data for further analysis and process. A typical data preparation pipeline includes normalization, filtering, aggregating, linearization, missing data elimination, feature extraction, and feature selection. The training phase includes the use of machine learning, ensemble learning, and deep learning techniques to develop the model that will be used for fault prediction. In the evaluation phase, the trained model is tested in its ability to predict the output by using various metrics such as accuracy, precision, recall, f-measure, confusion matrix, and area under the curve of receiver operating characteristics (AUCROC). In the prediction phase, probable machinery fault for previously unseen input data is produced by using the constructed model. Following that, the predicted output is presented to the users via a web/mobile application to provide guidance to the decision-makers. Finally, in the decision-making phase, the predicted result is utilized by managers for performing activities related to fault diagnosis, fault isolation, fault prognosis, fault detection, and exclusions.

## 3 Maintenance strategies

The word “maintenance” is referred to the procedure taken to preserve and repair a machine, component, structure, or building to its initial condition or to state where an effective functionality is obtained. The ultimate goal of maintenance is to operate the machine as expected, and prevent faults, breakdowns, or other problems that may endanger its reliability or performance. The maintenance procedure comprises various tasks including inspection, lubrication, cleaning, replacements, and repair. Because maintenance may be an expensive and time-consuming procedure, experts developed numerous maintenance strategies to lower such drawbacks.

Some significant strategies are preventive maintenance (PnM), predictive maintenance (PdM), corrective maintenance (CM), condition-based maintenance (CBM), reliability-centered maintenance (RCM), overhaul maintenance (OM), and run-to-failure maintenance (RFM). PnM focuses on regular inspections, cleaning, replacing, and other related tasks periodically to prevent failure [63]. PdM uses information obtained through sensors and other equipment to monitor the machinery and estimate the period when the replacement or repair is needed [64]. CM is based on fixing the part that is already malfunctioning or broken [[63], [65]]. CBM is similar to PdM as it also monitors the conditions of the components of the machine. However, CBM aims to give the exact time when the faulty part is needed to be replaced or fixed whereas PdM gives a future prediction regarding the time that the maintenance should start [66]. RCM focuses on the most essential parts of a machine and constitutes a maintenance schedule and strategy around those parts to guarantee their reliability [66]. OM is about taking the machine out of the order and conducting maintenance in which the machine parts are completely disassembled, inspected, cleaned, and reassembled [67]. Finally, the RFM strategy is based on zero proactive maintenance and waiting for the complete failure of the components [68].

Choosing the most suitable maintenance strategy is another challenging task. The selection of the correct method depends on the type, age, condition, fault type, and fault severity of the system or its component. In addition, the availability of replacement parts, safety and regulatory requirements, maintenance costs, skills of the maintenance technicians, and resources needed for maintenance are other essential parameters to adopting the appropriate maintenance strategy. In the industry, PdM is considered one of the most common strategies since it is cost-effective and reduces downtime by conducting a routine periodic check, which significantly lowers the possibility of unexpected repairs or breakdowns. PdM is also one of the most suitable approaches for Industry 4.0 [69,70] because PdM is a data-driven technique that can be easily implemented with the aspects of Industry 4.0 such as IIoT, AI, ML, and sensor technologies. Another appropriate strategy may be CBM, which also is a data-driven approach just like PdM. Because both strategies require data that helps to monitor the condition of the system/machine or its component, it is essential to understand fault diagnosis and prognosis procedures in monitoring the machines.

## 4 Machinery fault analysis strategies

Fault diagnosis (FD) and fault prognosis (FP) procedures refer to detecting, identifying, and assessing the faults or issues related to a machine to comprehend its current and future status. The main purpose of FD is to reveal the root cause of the fault and therefore, provide one to take the necessary actions to correct the malfunctioning. FD procedure involves detecting the condition of the machine, locating the faulty component, and finding the fault type if the machine is malfunctioning. These sequential sub-procedures are called fault detection (FDE), fault isolation or localization (FI), and fault identification (FID). Machine Learning-based fault diagnosis is generally conducted in six stages called data acquisition, data processing, feature extraction, model training, model assessment, and decision support. In data acquisition, signal or visual data is collected through sensors or cameras. Afterwards, the data is processed by signal or image processing techniques in the data processing stage to eliminate noise or any disruptive matter that adversely affects the characteristics of the data. In the feature extraction procedure, the time, frequency, time-frequency, or any other features of the processed or unprocessed data is extracted. If the model is untrained, then the feature sets are needed to be labeled for supervised learning-based and semi-supervised learning-based approaches. Later, the parameters of the intelligent model are tuned and the model is trained by considering the features as input and the labels as output. The trained model then is assessed by feeding it with unseen data. After the reliance and the performance of the model are proven, it may be implemented in a real-world setting as a decision-support mechanism for fault diagnosis. Unsupervised methods pursue a different strategy where the input data is unlabeled and the decision is made based on clustering. Regarding classification-based deep learning methods, it is not usually mandatory to perform the hand-crafted feature extraction stage since the model is generally capable of extracting the features automatically.

FDE can be expressed as the disclosure of an abnormal situation that may cause the machine not to work properly or even lead to an outage. This is also comprehended as the first step of the fault diagnosis procedure since the malfunction is identified yet, the kind, source, and severity of the malfunction are unknown. Fault detection can be conducted in various ways including visual or auditory inspections, monitoring the sensor data, statistical pattern control, and machine learning-based methods. Visual or auditory inspections are based on mostly work-experience subjective examinations in which the origin of a fault cannot be detected. Although can be related to fault detection, it may require expertise, training, and time for an effective inspection. Monitoring the sensor data provides a more in-depth approach to fault detection since the abnormality is detected by machine data such as vibration, temperature, pressure, etc. The sensor data is generally reliable and provides sufficient information about whether the machine is healthy or not. However, reading and interpreting the sensor data requires expertise. In addition, the data have to be collected from the healthy sensors that are properly mounted. Statistical pattern control employs statistical methods to find any changes in the trends or patterns in the sensor data to detect any abnormalities in the machine. This technique may reflect the issue of the machine in a simpler way than monitoring solely the sensor data. However, it still requires expertise due to the selection of the statistical metrics and reading such statistical data. Besides, early-stage abnormalities can easily go unnoticed since they may slightly impact the statistical patterns of a machine that operates normally. Machine learning-based methods utilize the sensor data or its characteristics obtained throughout image processing, signal processing, statistical feature extraction, etc. as input and pass them through a specified machine learning algorithm to find out whether the machine is healthy or not. Therefore, early-stage abnormalities may be detected in a fast and effective way. Although selecting an appropriate machine learning method and processing the data may cause a struggle, such techniques have the potential to provide the best and most precise results.

FI is expressed as the detection of a particular subsystem or component within a machine that causes an abnormality or failure. Following the fault detection procedure, it is one of the most essential steps in the fault diagnosis procedure since the exact location and component of the malfunctioning part is determined. Fault isolation can be performed by testing each component, tracing the output signal, and machine learning-based fault isolation methods. The component testing procedure is testing the functionality of the components to validate if they are working properly. As it can be estimated, testing each component can be time-consuming and expensive, which is undesirable in fault diagnosis procedures. To lower such consumption, substitution testing may be adopted in which the known healthy parts are swapped out with the existing components to observe whether the problem is solved or not. However, it is still an expensive and time taking procedure that is not generally preferred. Signal tracing is a testing method in which a signal is given during the test and it is traced to find the location where the signal does not behave normally. By employing the signal tracing technique the cost is significantly lowered and the malfunctioning component is usually located precisely. However, it requires expertise and time, especially for complex machines having numerous components. Machine learning-based fault isolation uses the visual or signal data to identify the abnormality and the abnormal component, including its location if the data acquisition devices are placed properly. As mentioned before, machine learning-based techniques require an appropriate algorithm selection, choosing and employing image or signal processing techniques, which are challenging tasks and require expertise.

FID refers to identifying the type of fault in a malfunctioning machine or component. This phase comprises the description of the fault instead of referring to the erroneous section as “faulty” only. Hence, it is a critical stage of the FD since the root cause of a fault is determined. By doing so, the necessary precautions and actions can be taken by the experts. FID can be employed by processing the vibration, acoustic, and temperature data of the machine since each fault type has a characteristic impact on such data. These data may be interpreted by the experts or provided to an AI-based advanced algorithm for an automated and effective FID procedure.

Fault Prognosis (FP) is known as predicting the severity of the faults that existed in a component of a machine that can be used for estimating the remaining useful life (RUL) or remaining life (RL) of the component. The procedure is conducted on the question of “How bad is the fault?”.

Thanks to FP, a possible catastrophic impending failure may be prevented. By providing an early warning, it is also possible to schedule maintenance or repairs, which may significantly reduce the downtime of the machine during maintenance. To conduct FP, the same methods as those implemented for FD are used. Apart from the FD, FP may also require a perspective of regression than classification. Therefore, it is necessary to focus more on the change in the trends of the measured data.

Developing effective fault diagnosis and prognosis approaches is vital in scheduling maintenance. Since each component has a unique impact on a rotating machine, it is essential to understand each component. Various kinds of faults may occur in the components specific to the rotating machines such as bearings, gear, induction motor, belt-drive mechanism, shafts, fans, turbine blades, etc. Hence, developing component-specific and fault-specific diagnosis and prognosis strategies or methods is a key procedure to take in time and correct actions.

## 5 Fault diagnosis and prognosis in rotating machinery

**Fig. 4:**
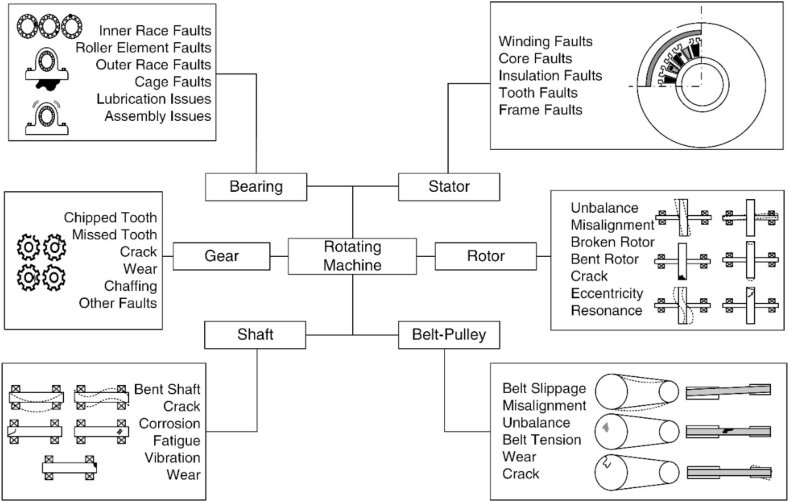
The components and faults in the rotating machine.

A rotating machine refers to a system where the components rotate around an axis to generate mechanical energy for numerous purposes. Such machines are utilized in pumping fluids, turbines, generators, fans, and compressors. The fault diagnosis and prognosis procedures regarding a rotating machine are generally conducted on its components. Fig. 4 shows the parts of the rotating machinery including the fault types that may occur.

**Fig. 5:**
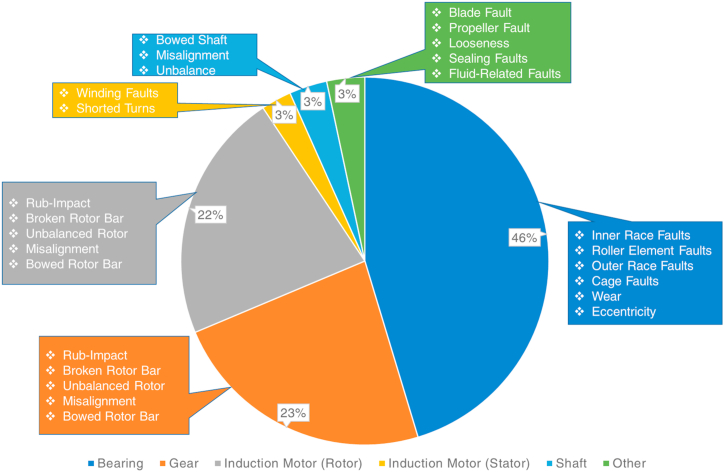
The percentile distribution of the considered rotating machine.

In the last ten years, numerous studies related to intelligent fault diagnosis and prognosis (IFDP) of rotating machines are conducted and published in the literature. Fig. 5 shows the percentile distribution regarding the considered published studies and the considered machine component.

**Fig. 6:**
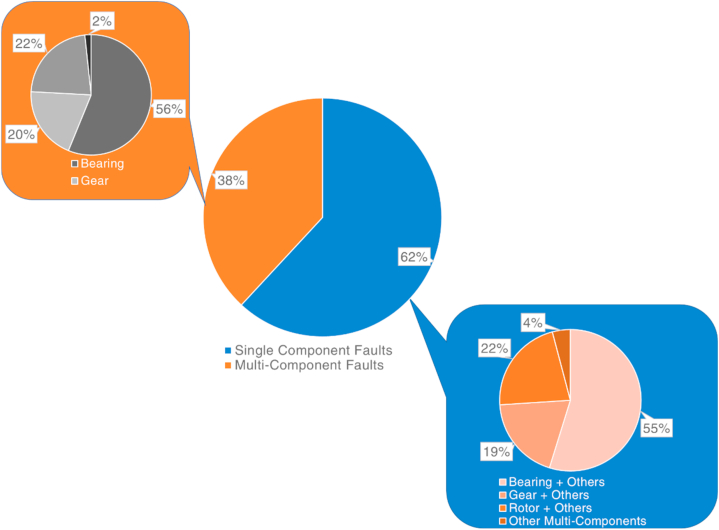
The ratio of IFDP methods build for single component faults and multi-component faults.

**Fig. 7:**
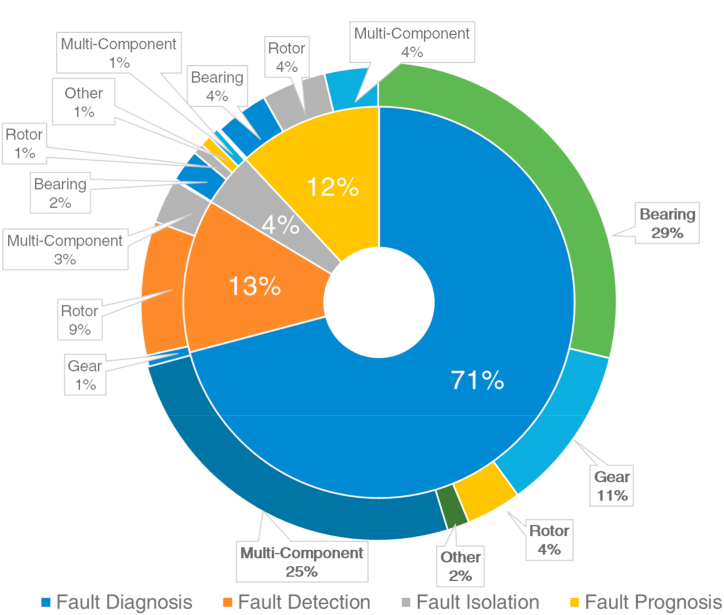
The percentile distribution of the considered strategies regarding IFDP of rotating machines.

It is seen in Fig. 5 that the majority of the studies are considered bearing faults [[71], [72], [73], [74], [75]] followed by rotor faults [[76], [77], [78], [79], [80]], gear faults [[81], [82], [83], [84], [85]], shaft faults [21,46,71,72,86], stator [7,25,87], and other component faults [28,[88], [89], [90]]. The pie chart shown in Fig. 5 comprises studies that consider the faults of a single component and the faults of multi-components. As seen in Fig. 6, most studies developed an intelligent model to find the fault types of a single component. However, there are a considerable amount of studies that also considered multi-component faults to build IFDP models for rotating machines [[91], [92], [93], [94], [95], [96], [97], [98], [99], [100], [101]]. Fig. 7 shows the percentiles of the considered fault analysis and intelligent model development strategies regarding FD, FDE, FP, and FI. Among those studies, it is seen that the majority pursued to constitute an FD-based intelligent model, followed by FDE, FP, and FI models.

### 5.1 Bearing faults

Bearings are the mechanical parts in rotating machines that are used to guide and support the moving components. They have a key role in an effective process by reducing friction, supporting the rotating components, and providing a consistent and smooth movement. Faults in bearing may occur due to various reasons including excessive loading, poor bearing selection, faulty installation, lack of maintenance, excessive vibration, etc. The most common bearing faults are related to the inner race, rolling element, cage, and outer race of the bearing. Besides, assembly and lubrication issues may cause wear, excessive vibration, and oil leakage [102].

**Fig. 8:**
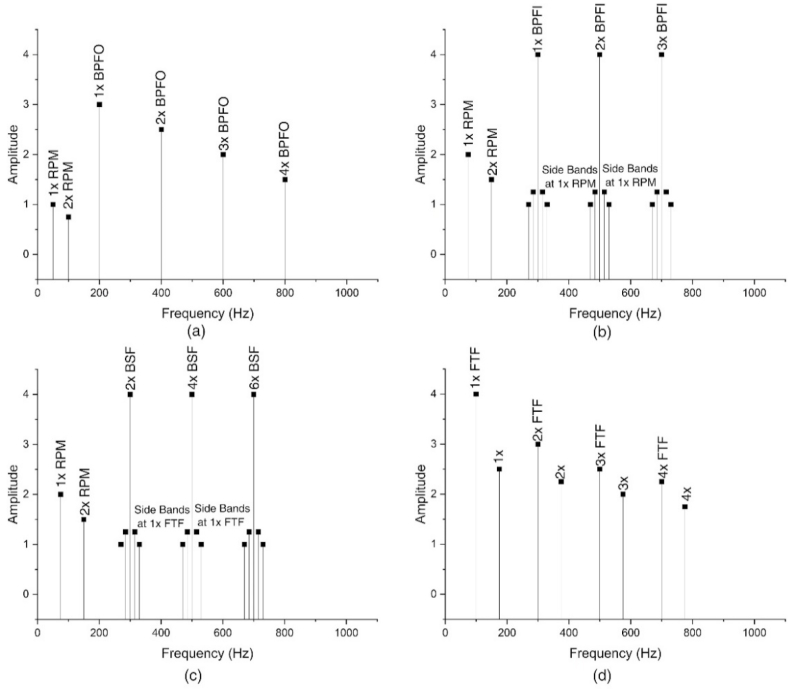
The frequency spectrum of (a) outer ring fault, (b) inner ring fault, (c) ball fault, and (d) cage fault.

The faults manifest themselves in characteristic frequencies. Ball pass frequency outer (BPFO) refers to the outer race faults whereas ball pass frequency inner (BPFI) is an indicator for inner race faults. Regarding the rolling element faults, ball spin frequency (BSF) is used. Finally, the cage faults are referred to as fundamental train frequency (FTF). If the bearings are not replaced when those frequencies appeared, the following stage will affect the 1× RPM amplitude and make the BFPO, BPFI, BSF, and FTF disappear. At frequencies close to and higher than 30 k Hz, random vibrations are observed. These characteristic fault frequencies are calculated as [102].

(1) $$BPFO=\frac{n}{2}\;\left(1-\frac{d_{B}}{d_{P}}\cos\;\theta \right)\frac{rpm}{60}$$

(2) $$BPFI=\frac{n}{2}\;\left(1+\frac{d_{B}}{d_{P}}\cos\;\theta \right)\frac{rpm}{60}$$

(3) $$BSF=\frac{d_{P}}{2d_{B}}\left(1-{\left(\frac{d_{B}}{d_{P}}\right)}^{2}\cos^{2}\;\theta \right)\frac{rpm}{60}$$

(4) $$FTF=\frac{1}{2}\left(1-\frac{d_{B}}{d_{P}}\cos\;\theta \right)\frac{rpm}{60}$$

where *n* is the number of balls, θ is the contact angle, *d*_*B*_ and *d*_*P*_ are the ball diameter and pitch diameter, respectively. The frequency spectrums of those characteristic frequencies are shown in Fig. 8 [102].

**Fig. 9:**
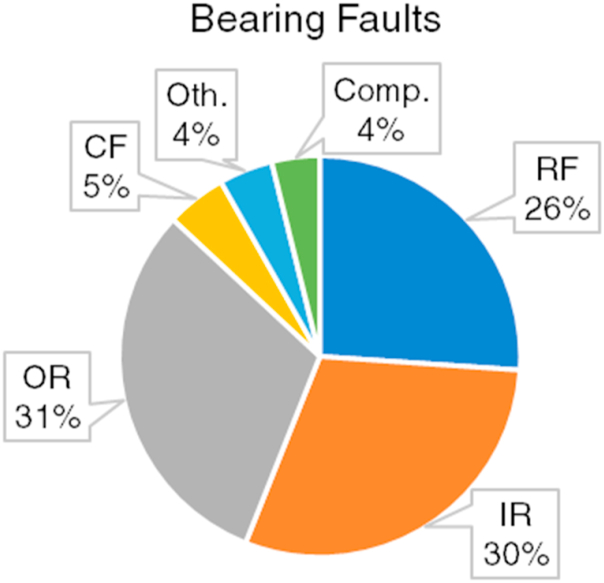
Percentile distribution of the considered bearing fault types.

Regarding the intelligent fault diagnosis and prognosis (IFDP) of bearing faults, numerous studies are presented in the last decade. Table 1 refers to the studies related to the intelligent bearing fault diagnosis and prognosis. The abbreviations of the fault diagnosis and prognosis procedures in Table 1 are as follows. FD: Fault Diagnosis, FDE: Fault Detection, FI: Fault Isolation (Fault Localization), FP: Fault Prognosis. The abbreviations of the bearing fault types in Table 1 are; RF: Rolling Element Fault, IR: Inner Race Fault, OR: Outer Race Fault, CAF: Cage Fault, Comp.: Compound Faults, Oth.: Other Faults, (c): Classification. Fig. 9 shows the percentile distribution of the bearing fault types investigated within the scope of the studies given in Table 1.

**Table 1:** Intelligent fault diagnosis and prognosis studies related to bearing faults.

Studies	Year	Procedures				Fault Types				
		FD	FDE	FI	FP	RF	IR	OR	CAF	Comp./Oth.
[16]	2013	X					X	X		
[71]	2014	X						X		
[6]	2013		X							Ageing
[124]	2014	X			X(c)		X	X		
[39]	2014	X			X(c)	X	X	X		
[91]	2014	X			X(c)	X	X	X		
[92]	2015	X			X(c)	X	X	X		
[93]	2015	X				X	X	X		House Eccentricity
[123]	2015	X				X	X	X		Retainer Fault
[72]	2015	X					X	X		Wear, Lack of Bearing
[94]	2016	X						X		Lack of Lubrication
[7]	2016	X		X		X				
[87]	2016	X					X			
[41]	2017	X			X(c)	X	X	X		
[95]	2017	X				X	X	X		
[118]	2017	X				X	X	X		
[48]	2018	X		X		X	X	X		
[74]	2018	X				X	X	X		IR + OR
[97]	2018	X				X	X	X		House Eccentricity
[100]	2018	X				X	X	X	X	
[47]	2019	X					X	X		
[23]	2019	X					X	X	X	
[126]	2019	X				X	X	X		
[127]	2019	X			X(c)	X	X	X		
[22]	2019	X								Wear
[40]	2019	X				X		X		Gear + Bearing, Gear + Bearing + Looseness
[38]	2020	X		X		X	X	X		
[109]	2020	X		X		X	X	X		
[110]	2020	X				X	X	X		
[111]	2020	X			X(c)	X	X	X		IR + OR, RF + OR
[114]	2020	X				X	X	X	X	
[115]	2020	X				X	X	X		
[37]	2021	X				X	X	X		
[32]	2021	X				X	X	X	X	LB
[35]	2021	X				X	X	X		
[106]	2021	X				X	X	X		BF + IR + OR
[107]	2021	X			X(c)		X	X		IR + OR
[108]	2022	X				X	X	X	X	
[10]	2022	X					X	X		IR + OR
[45]	2022	X				X	X	X	X	
[103]	2022	X				X	X	X		
[101]	2022		X			X	X			
[75]	2022	X					X	X	X	
[105]	2022	X				X	X	X		
[15]	2022	X	X			X	X	X	X	
[11]	2023	X				X		X	X	Rotor + Bearing
[125]	2023	X					X	X

As seen from Fig. 9, the majority of those studies are focused on the rolling faults [[103], [104], [105], [106]], inner race [[107], [108], [109], [110], [111], [112], [113], [114], [115], [116], [117], [118]], and outer race defects [[119], [120], [121], [122], [123], [124], [125], [126], [127]]. On the other hand, cage faults are also examined by the researchers, but not much as the ball, inner race, and outer race faults. The remaining faults such as house eccentricity or lubrication issues constitute the minority of the literature. Another significant condition is the compound fault situation where multiple different faults take place at the same time. The literature comprises a few studies related to this condition regarding bearing faults. Besides, some studies examined compound faults that include the same or different component faults in addition to the bearing faults [10,40,97,106,107,111].

The studies given in Table 1 mostly used publicly available benchmark datasets. Most of the studies considered benchmark datasets, especially those in which ball, inner race, and outer race faults include. In addition, the dataset also comprises different fault locations regarding the outer race faults. The measurements of the dataset are conducted considering four motor loads. Apart from this dataset, various studies use the data from their setup or the industry [6,7,16,22,23,32,71,74,87,92,94,95,99,107,113,114,116,124].

Some studies related to the intelligent bearing fault diagnosis and prognosis are presented as follows. Li et al. [93] proposed a support vector classification-based approach to identify bearing faults. For this purpose, they considered two gearbox setups where different gear and bearing faults exist. Regarding the bearing faults, the setup includes IR, OR, RF, and House Eccentricity (HC) faults. Although they adopted an FD procedure, the model performance on the fault identification is not clear because only overall model accuracy is considered. Shoaib et al. [41] employed a deep learning-based approach for bearing FD and FP. They considered RF, IR, and OR faults. As performance metrics, only overall model accuracy is considered. Although overall accuracy is an essential indicator for model performance, it is not sufficient for FD procedure because overall accuracy does not include the fault-specific performance of a proposed approach, which is essential especially if multiple types of faults are asked to be found. The study also comprises a classification-based FP approach with an accuracy higher than 96.50% regarding each fault severity. Zhang et al. [48] used a presumption-based Naive Bayes approach for the FD and FI of bearings. They employed RF, IR, and OR faults of bearings. They considered both accuracy and confusion matrices for FD and FI. The usage of the confusion matrix enabled them to provide the fault-specific performance of their proposed model for the FD procedure. Regarding FI, they considered OR faults that exist at the center at @6:00, orthogonal at @3:00, and opposite at @12:00 locations of the outer race of the bearing. The FI procedure is conducted in combination with the FD procedure. In other words, they considered each location of OR fault as a distinct labeled case and built the model with that assumption. Hence, instead of four classes including normal case (NC), IR, RF, OR; six cases (NC, IR, RF, OR@3:00, OR@6:00, and OR@12:00) are considered. The overall accuracy of their proposed approach is 99.17% whereas the fault-specific prediction accuracy differs between 97.50% and 100.00%. Sanchez et al. [97] considered five different datasets for the FD of bearings. Four bearing faults, RF, IR, OR, and HC are predicted by two machine learning-based techniques. To measure the FD performance of their proposed approach, they only considered overall model accuracy for each wavelet and feature type. The highest overall FD accuracy is obtained by 83.0%. Guo et al. [38] used a deep learning-based approach for FD and FI of bearings. They considered RF, IR, and OR faults that existed on a simulator setup. According to their experimental results, they only considered only overall model accuracy values for both FD and FI. They obtained an overall accuracy of 96.04% for FD and 99.93% for FI of bearings. Choudary et al. [32] employed a deep learning-based method for bearing FD of rotating machines. They considered bearings having RF, IR, OR, CAF, and lack of lubrication (LB) faults. They presented the model performance of their proposed approach by using overall and fault-specific accuracy, precision, recall, F-score values, and confusion matrix. According to the experimental results, their proposed approach makes predictions with an overall accuracy of 99.80%. Considering the fault-specific performance of the proposed model, the accuracy values change between 97.05% and 100.00%. Presenting precision, recall, and F1 scores indicate the sensitivity and robustness of their proposed approach. These are also essential metrics that illustrate how well the model makes predictions when it deals with related unseen data.

As seen in Table 1, most of the studies focused on FD rather than FDE, FI, or FP. In general, a complete FD procedure comprises FDE and FI. Hence, all FD studies are also related to FDE and FI in some way. On the other hand, FP is about the fault severity and the remaining useful life (RUL) of the component. There are only a few studies that are interested in FP for bearings [29,39,41,111,125]. Besides, most of those studies adopted a classification-based strategy for FP, which may give a fallacious idea regarding the performance of an intelligent model. For FP procedures, confusion matrices should be also considered. Acquiring a high accuracy value for each severity is important, but considering a classification-based FP procedure may not reflect the true performance of a proposed model since severity values can vary infinitely unlike labeled fault types. For instance, consider a dataset that comprises three IR crack lengths, 0.01 mm, 0.02 mm, and 0.03 mm. Building an intelligent fault prognosis model will be labeled with those values if a classification-based strategy is employed. An accurate model will predict the unseen data that includes 0.01 mm, 0.02 mm, or 0.03 mm crack length. On the other hand, it is unknown how it will classify the data which includes 0.015 mm, 0.025 mm, etc. The model may predict the crack length of 0.015 mm as 0.01 mm or 0.02 mm. Such situations make the classification-based FP strategies generally unreliable and inappropriate for severity prediction and/or RUL. Regarding performance metrics, numerous studies focused on only accuracy [31,37,39,41,45,47,50,73,104,112,119] and discarded the other metrics such as confusion matrix, precision, and recall. The omission of the confusion matrix in the FD procedure makes it unable to interpret fault-specific model performance. Hence, the prediction ability of a proposed approach remains unknown for a specific type of fault, which endangers the reliability of a model. The confusion matrix also enables the evaluation of other metrics such as precision and recall which are the indicators of the sensitivity and robustness of an intelligent model. Those metrics should also be presented regarding the overall and fault-specific performance of the intelligent FD technique. Considering precision and recall illustrates the performance of the model for unseen related data. Some studies considered confusion matrices and/or precision-recall [16,26,47,51,55,129], yet they constitute the minority of the literature.

According to the studies given in Tables 1 and it is seen that regression-based strategy for FP procedures is lacking in the current literature. In addition, confusion matrix, precision, and recall metrics are mostly not considered to reflect the fault-specific performance, sensitivity, and robustness of a proposed intelligent FD method. Regarding the fault type percentiles shown in Fig. 9, it is seen that the majority of the studies focused on only four types of bearing faults whereas other faults such as house eccentricity, aging, journal bearing faults, oil issues, wear, and clearance problems are barely investigated [6,22,31,72,93,123]. Besides, compound faults are rarely considered [10,40,74,106,107], yet they are essential in FD and FP procedures of rotating machines.

### 5.2 Gear faults

**Fig. 10:**
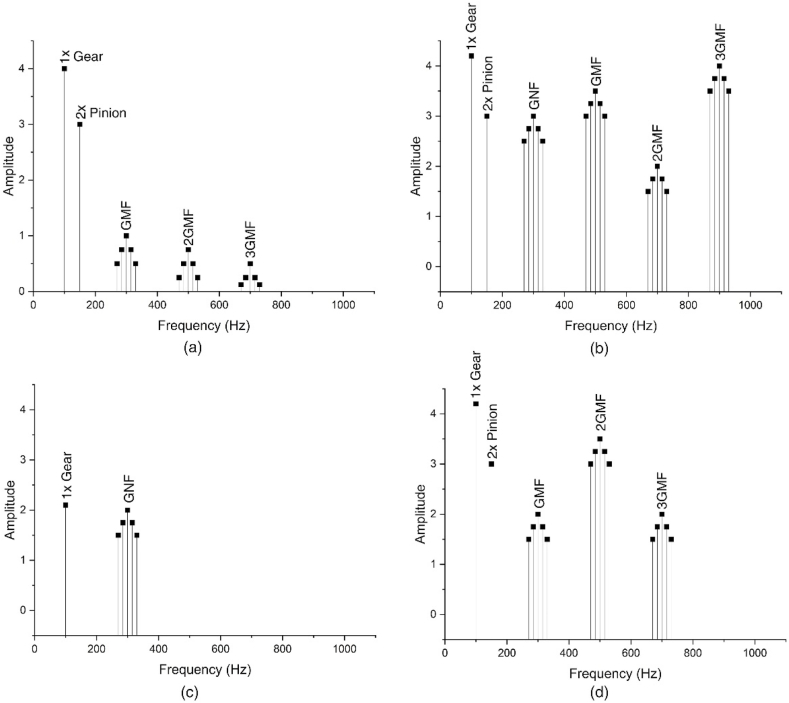
The frequency spectrum of (a) healthy gear, (b) tooth wear damage, (c) cracked/broken tooth, and (d) gear misalignment.

Gears are one of the most critical parts of rotating machines because they transmit power and motion between rotating components. They are shaped and sized differently in accordance with the needs. Gear faults may occur due to excessive loading, faulty installation, improper or lack of lubrication, fatigue, corrosion, or contamination by dust, dirt, or any material that can harm. Some gear faults are tooth wear, broken or cracked tooth, chipped tooth, gear misalignment, and eccentricity. Just like bearing faults, gear faults show themselves uniquely in the frequency spectrum. Fig. 10 shows the frequency spectrum of some gear faults [102].

Diagnosis and prognosis of the gear faults attracted researchers from the past to the present. In the scope of the IFDP, various approaches are proposed regarding different gear fault types. Table 2 refers to the studies related to the intelligent bearing fault diagnosis and prognosis. The abbreviations of the gearing fault types in Table 2 are as follows. *CT: Chipped Tooth, IR: Inner Race Fault, BT: Broken Tooth, MT: Missed Tooth, PT: Pitting, CRC: Crack, WT: Worn Tooth, Comp.: Compound Faults, Oth.: Other Faults, (c): Classification*.

**Table 2:** Intelligent fault diagnosis and prognosis studies related to gear faults.

Studies	Year	Procedures				Fault Types						
		FD	FDE	FI	FP	CT	BT	MT	PT	CRC	WT	Comp./Oth.
[91]	2014	X			X(c)	X		X				
[92]	2015	X			X(c)	X	X		X			Chafing, Face Wear
[93]	2015	x				X		X	X		X	Chaffing
[81]	2015	X					X		X			Face Wear, Misallignment
[24]	2016	X				X			X	X	X	Chafing
[17]	2016	X					X				X	
[95]	2017	X			X(c)					X		
[96]	2017	X										Unknown
[43]	2017	X				X			X	X	X	Chafing, Weak Root
[82]	2017	x							X	X		
[74]	2018	X			X(c)		X					
[97]	2018	X					X			X	X	Chafing, Misalignment
[98]	2018	X					X		X			
[42]	2018	X				X	X	X			X	
[21]	2018	X				X	X					Eccentricity
[99]	2018	X					X		X			Surface Fatigue
[131]	2018	X		X						X		
[24]	2019	X			X(c)					X		
[130]	2019	X				X		X		X	X	
[40]	2019	X				X	X			X		BT + CT,Gear + Looseness, Gear + Looseness + Bearing, Gear + Bearing
[129]	2020	X					X	X		X	X	
[113]	2020	X				X						
[8]	2020	X										Unknown
[83]	2020	X							X	X	X	Chafing
[9]	2020	X				X		X	X			Eccentricity, Abrasion
[107]	2021	X			X(c)		X					
[13]	2021		X									Unknown
[27]	2021	X				X		X		X	X	
[51]	2021	X					X		X		X	
[128]	2021	X						X		X	X	Gnash
[85]	2021		X				X					
[101]	2022		X								X	
[75]	2022	X								X		
[84]	2022	X								X		Spalling

**Fig. 11:**
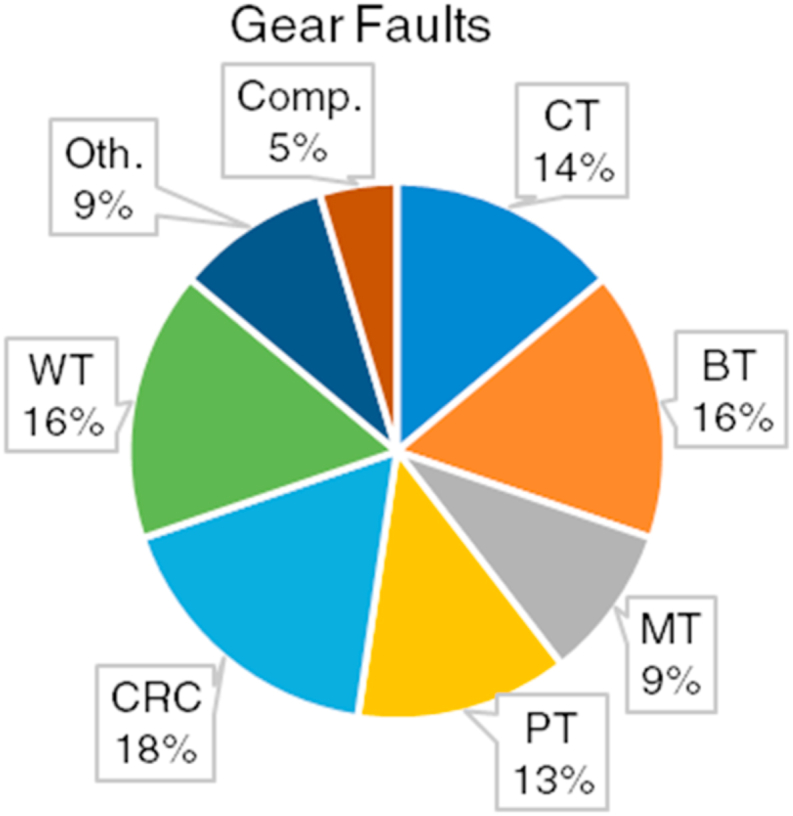
Percentile distribution of the considered gear fault types.

Fig. 11 shows the percentile distribution of the bearing fault types investigated within the scope of the studies given in Table 2.

As seen in Fig. 11, an almost equal distribution is observed considering CT, WT, BT, CRC, and PT faults [[128], [129], [130], [131]]. The MT fault is slightly less investigated than those faults. Gear faults like chafing, surface wear, gnash, eccentricity, and spalling are rarely examined [9,81,83,84,128]. All these faults constitute only 4% of the total studies. The compound faults among gear faults are also barely investigated. Among all the studies taken into account, only one study measured the performance of their proposed approach on compound faults considering gear compound faults and gear-other component compound faults [40].

As seen in Table 2, most of the studies focused on the FD procedure rather than FDE, FI, or FP. On the other hand, conducting a complete FD also means performing FDE and FI. Only about 17% of the studies are focused on classification-based FP procedures. Some studies related to IFDP regarding gear faults are presented as follows. Chen et al. [92] adopted a deep learning-based approach for FD and FP of the gearbox where various gear and bearing faults exist. Regarding the gearing faults, face wear (FW), chafing (CH), BT, PT, and CT. They evaluated their proposed approach by considering accuracy and confusion matrix metrics. In addition, mean and median statistics of the accuracy values are obtained from the confusion matrix. FD and FP procedures are considered as a whole, meaning that the same faults having different severity values are treated as labeled unique fault classes. The mean overall accuracy of the proposed model is 96.8%. Regarding fault-specific results, the accuracy values differ between 91.4% and 98.9%. Although precision and recall metrics are not given, they can be evaluated through a confusion matrix. Chemseddine et al. [42] used a neural network approach for gear FD of a gearbox. The gears in the gearbox contain CRC, CT, MT, and WT faults. The performance of their model is measured by considering only the overall model accuracy metric regarding the five sequences that they performed. They obtained an overall accuracy of 99.36% in classifying gear faults. Azamfar et al. [9] proposed a deep learning-based technique for gear FD. For this purpose, they used the data obtained from a test simulator where eccentricity (ECC), PT, MT, CT, and abrasion (ABR) faults exist. The model performance is reflected by the accuracy and confusion matrix. The study also comprises FP procedures regarding ABR fault. Similar to various studies, a classification-based approach is pursued by considering different ABR severity values as unique fault classes. Shi et al. [84] considered the sun-planet gear mechanism for FD of gear faults. They used a deep learning-based approach for gears having CRC and spalling (SP) faults. They performed a complete FD that includes fault detection, fault type identification, and fault location. In addition, they also considered finding the fault direction. To assess the model, they considered both overall model accuracy and confusion matrix metrics. Hence, fault-specific model performance can be evaluated. On the other hand, the fault-specific results are not summarized, yet they can be evaluated from the confusion matrices.

As mentioned in bearing faults, the studies related to the FP of gear faults pursued a classification-based approach [91,107,127]. Although classification-based FP may give an idea about the performance of the proposed approach, it may misdirect one as the developed model receives an input that indicates a severity value close to the mean value of two severity classes introduced to the model. Regarding FD procedure, various studies generally focused on overall model accuracy [8,21,24,27,40,42,43,97,129], and a few considered confusion matrix, precision, and recall [85,101,107]. The gear faults considered in related studies are diverse since numerous faulty gear cases are examined. On the other hand, the literature lacks compound gear faults or compound faults including gear faults and other rotating machine faults.

### 5.3 Rotor faults

**Fig. 12:**
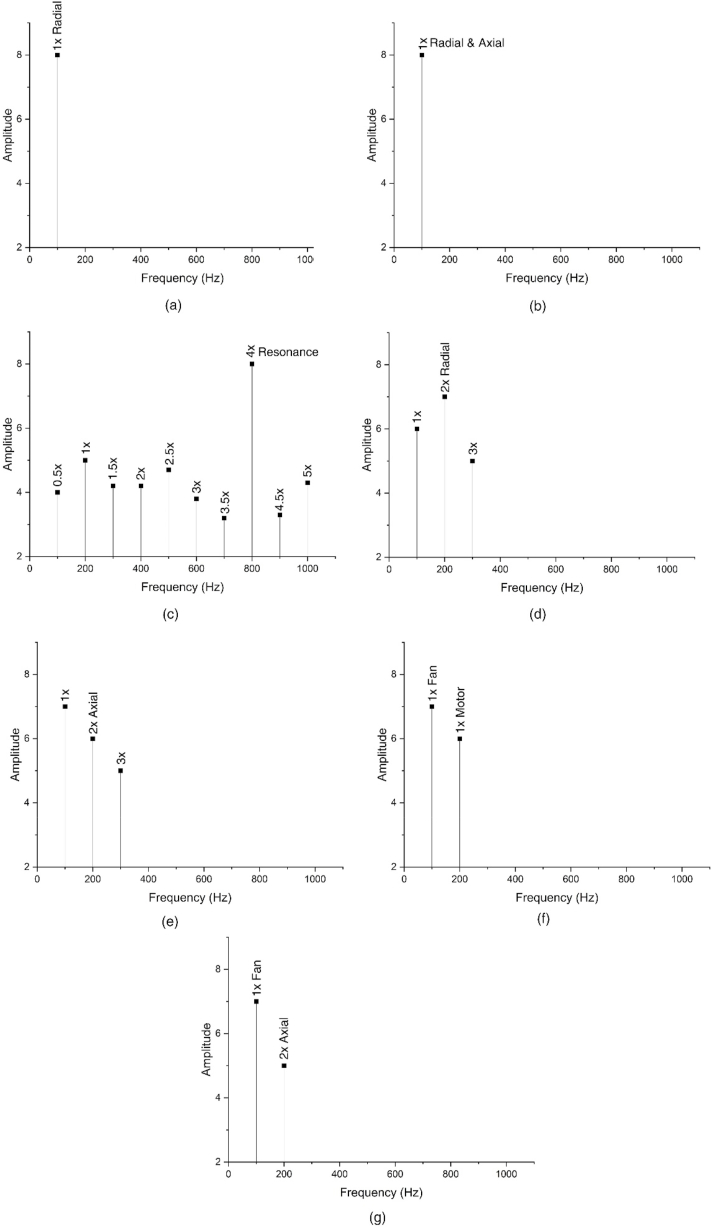
The frequency spectrum of (a) unbalance fault (force, dynamic, couple), (b) unbalance fault (overhung rotor), (c) rub-impact fault, (d) horizontal misalignment, (e) angular misalignment, (e) eccentric rotor, and (f) bent shaft.

The rotor is used for producing the rotational motion that drives or is driven by a machine. It is surrounded by a stator, which is a stationary part that provides electric current to rotate the rotor. Rotor faults occur due to several reasons including manufacturing and maintenance issues, exposure to environmental factors, excessive loading, faulty installation, aging, and operating conditions. The types of rotor faults are broken rotor bars, unbalanced rotor, misaligned rotor bars, rub-impact faults, corrosion, and electrical faults. Each kind of fault has a unique impact on the rotating machine data that includes vibration, temperature, pressure, current, etc. The illustration of the impact of the rotor-related faults on the frequency spectrum is shown in Fig. 12 [102].

**Fig. 13:**
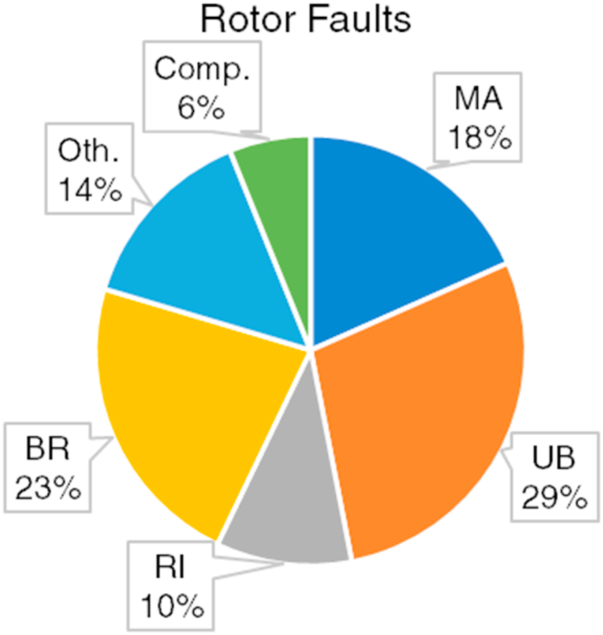
Percentile distribution of the considered rotor fault types.

Table 3 presents the studies related to the intelligent rotor fault diagnosis and prognosis of rotating machines. The abbreviations of the rotor fault types in Table 3 are; *MA: Misalignment, UB: Unbalance, RI: Rub-Impact, BR: Broken Rotor, BWR: Bowed Rotor, ECC.: Eccentricity, DICB: Damaged Impeller and Cover Board, OW: Oil Whirl, DR: Damaged Rotor, RWF: Rotor Winding Fault, Comp.: Compound Faults, Oth.: Other Faults, (c): Classification.* Fig. 13 shows the percentile distribution of the bearing fault types investigated within the scope of the studies given in Table 3.

**Table 3:** Intelligent fault diagnosis and prognosis studies related to rotor faults.

Studies	Year	Procedures				Fault Types				
		FD	FDE	FI	FP	MA	UB	RI	BR	Comp./Oth.
[136]	2013		X		X(c)		X			
[25]	2013	X								ECC.
[6]	2013		X						X	
[30]	2013		X		X(c)		X			
[71]	2014	X								BWR
[97]	2014	X			X(c)					CR
[76]	2015	X				X	X	X		
[138]	2015	X		X		X	X			CR
[94]	2016	X					X			
[137]	2016		X		X(c)		X			
[7]	2016	X		X					X	
[87]	2016	X					X		X	BWR
[77]	2017	X	X						X	
[140]	2017		X		X(c)				X	
[96]	2017	X				X				
[135]	2018	X				X	X	X		
[26]	2018		X						X	
[78]	2018		X						X	
[100]	2018	X				X	X			
[49]	2019	X								DICB
[133]	2019	X				X	X	X		OW
[134]	2019		X						X	
[22]	2019	X								DR
[40]	2019	X				X	X			Rotor + Gear Faults
[79]	2019		X					X		
[114]	2020	X					X			Rotor + Bearing Faults
[115]	2020	X								DR
[12]	2021	X			X(c)				X	
[132]	2021	X				X	X			
[34]	2021	X						X		
[33]	2022		X						X	
[141]	2022		X		X(r)					RWF
[80]	2022		X						X	
[11]	2023	X				X	X			Rotor + Bearing Faults
[139]	2023		X		X(c)					CR

It is seen from Table 3 that compared with the gear and bearing faults, there are more studies in percentile for rotors that focused on the FP procedures. Besides, although numerous studies comprise FD of rotor faults, there are a considerable amount of FDE studies. The FDE studies are based on fault detection and do not aim to identify the type of faults. Since there is only a healthy and faulty condition of the component, the importance of the considered defects is only related to the level of distinguishability between faulty and healthy states. Regarding FDE procedures, RI faults are considered more than the other rotor faults. In Table 3, some studies are referred to as FD studies that have a single rotor fault. This is because these studies are focused on not only rotor faults but also other components and assessed their proposed approaches by considering all kinds of faults. Some studies related to the IFDP of rotors are presented as follows. Lu et al. [79] employed a machine learning-based method for the FD of rotor faults. They considered UB, MA, and RI faults that existed in a rotating machinery setup. Although they used accuracy as the sole performance metric, they also present fault-specific results regarding the assessment of their proposed approach. They obtained a perfect score for all kinds of conditions and fault types of the rotor. Zgarni et al. [78] performed an intelligent FDE procedure regarding the BR fault of the rotor. To evaluate the performance of their proposed method, they considered ROC curves, F1 score, and false faction classification (FFC) score. Obtaining an F1 score close to 1 indicates a precise and sensitive model. FFC score is another metric that indicates the average false predictions made by the model. Hence, a lower FFC means more accurate results. According to their experimental results, the highest metrics are obtained as 1.0 for F1 and 0.0 for FFC. Since intelligent FDE procedures are binary classification problems, a confusion matrix may not be essentially required. Xiang et al. [139] adopted a deep transfer learning-based approach for rotor FDE and FP where cracked rotor fault (CF) having two severity values is considered. Although it is an FDE problem, the confusion matrix is considered a performance metric in addition to overall accuracy. The inclusion of the confusion matrix is because of the FP procedure where two crack severities are identified in a classification-based approach. According to their experimental results, they obtained an average model accuracy of 92.67% and perfectly distinguish one crack from another.

As seen in Table 3, there are plenty of studies related to the FP of rotor faults. However, similar to bearing and gear faults, almost all of those are classification-based approaches. Hence, regression-based strategies for FP procedures [141] constitute an essential gap in the literature regarding rotor faults. The performance metrics of the IFDP studies related to rotor faults mostly include only overall model accuracy, which may not be sufficient to understand the fault-specific performance of a proposed technique. As seen in Fig. 13, a significant variety of rotor faults are covered. On the other hand, these rotor faults are mostly examined regarding single-fault occurrences where it is assumed that a single fault exists in the system. Although there are studies that investigated compound fault conditions [11,40,114], they considered a rotor fault that occurred at the same time as a fault of another component such as a bearing or gear.

### 5.4 Other faults

Rotating machines comprise various other faults that occur on the bearing, gear, or rotor including belt-pulley faults, fan or turbine faults, looseness, shaft faults, etc. As seen in Fig. 5, these faults constitute the minority of the studies related to IFDP. Table 4 presents the IFDP studies of the different faults that may occur in rotating machines.

**Table 4:** Intelligent fault diagnosis and prognosis studies related to faults of other components in rotating machine.

Studies	Year	Procedures				Component	Fault Types
		FD	FDE	FI	FP		
[22]	2013	X				Stator	Stator Winding
[67]	2014	X				Shaft	Bowed
[85]	2014	X		X		Belt-pulley	Belt Looseness, Belt Damage, Pulley Damage
[68]	2015	X				Shaft	Bowed Shaft
[7]	2016	X		X		Stator	Shorted Turns
[83]	2016	X				Stator	Shorted Turns
[82]	2017	X				Shaft	Misalignment, Imbalance, Misalignment + Imbalance
[78]	2017	X				Shaft	Shaft Crack
[18]	2018	X				Shaft	Imbalance, Damaged Shaft
[131]	2018	X				Bearing Block	Looseness
[43]	2019		X			Shaft	Misalignment
[37]	2019	X				Shaft	Looseness, Looseness + Gear Fault
[128]	2021	X				Couplings	Looseness
[15]	2022	X	X			Turbine Blade	Blade Fault
[25]	2023		X			Turbine Blade, Fastener	Blade damage, Bolt Damage

According to Table 4, the majority of the studies are related to the FD procedure of shaft and stator faults. The faults of the remained components such as the belt-pulley, turbine blade, or fasteners of the rotating machine are rarely investigated. There are also a few studies related to the looseness faults that may occur in different parts of the rotating machines [40,132,135]. Similar to the studies related to bearing, gear, or rotor faults only a few studies take confusion matrix, precision, recall, or any other metrics in FD procedures of rotating machines. Most of the studies assessed their intelligent model based on the overall accuracy of fault detection and fault identification in rotating machines [15,21,25,40,46,88]. In addition, the majority of the studies are focused on single fault occurrences rather than compound faults [40,86].

## 6 AI-based approaches in fault diagnosis and prognostics of rotating machines

### 6.1 Data sources

In the IFDP of rotating machines, various kinds of data sources and data acquisition methods are used to constitute an intelligent model for FD and FP. Each fault has a unique impact on the machine monitoring data in different ways. Hence, it is essential to choose a suitable data source and data acquisition technique for the faulty conditions of a rotating machine.

The most common data sources are as follows. *Visual Data* comprises image data obtained through high-resolution cameras, optical devices, or images derived from a signal. Visual data may include thermal images [32,114], x-ray images [143,144], acoustic signals [11,23,24], or vibration data [75,85,106] or their processed form including spectrogram [145,146], waveforms [147], etc that usually reflects the characteristics of the healthy and faulty behavior of a component of a machine. *Acoustic Data* includes the sound or noise that is produced by a rotating machine that differs when operating in a healthy and faulty condition. Acoustic data is gathered via a microphone, acoustic emission sensors, and accelerometer [11,23,24] to conduct an effective FD and FP procedure for rotating machines. *Vibration/Kinematic Data* is constituted by the dynamic behavior of a rotating machine such as vibration, acceleration, or velocity. Vibration/Kinematic Data can be gathered by accelerometers, tachometers, and gyroscopes [11,30,74,84,91,96] to determine the condition of the rotating machinery. There are also other data sources and data acquisition methods for IFDP of rotating machines including the measurement of feeding currents by transducers, magnetic field by magnetometers, EMG, and pressure/depth values by depth sensors [9,27,47,97,141]. The selection of the data source and data acquisition method depends on cost, effectiveness, real-time adaptability, environmental conditions, robustness, sensitivity, installation, and maintenance complexity.

**Fig. 14:**
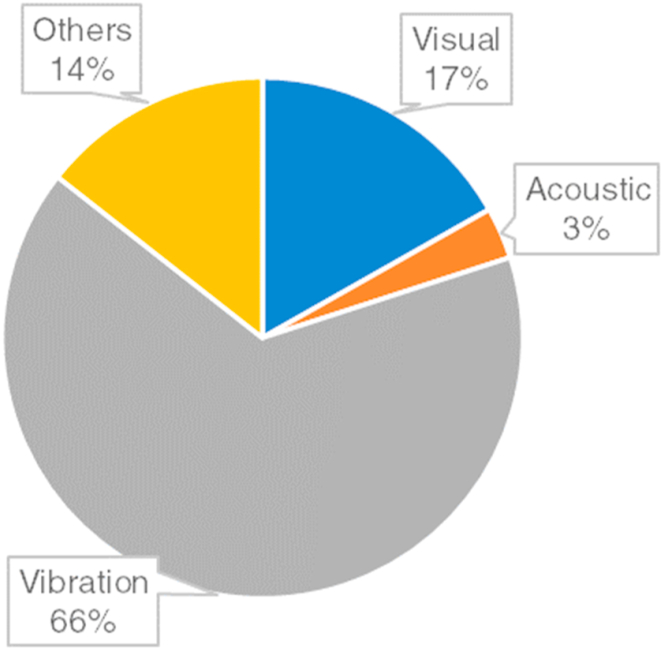
The considered type of data source distribution for IFDP of rotating machines.

As seen in Fig. 14, vibration data is generally used for the IFDP of the bearing, gear, rotor, and other components. Visual/image data are also considered for mostly FD of rotating machine components. However, these data are broadly used for bearings and rotors. In addition, visual/image data are frequently used for FD procedures whereas a few studies used them for FDE, FI, and FP. Acoustic signals are also used, yet such studies are lesser than the other data sources regarding the FD of rotating machines. Apart from those data sources, some studies used current signals, EMG, SCADA data, temperature, torque, pressure, and velocity for the IFDP of rotating machine components.

Table 5 shows the studies and the data sources used for FD, FDE, FI, and FP procedures of rotating machine components. The abbreviations given in the table are; *ES: Electric Signal, TQ: Torque, PR: Pressue, TM: Tachometer*. Some of those are briefly presented as follows.

**Table 5:** Intelligent fault diagnosis and prognosis studies and considered data source types.

Studies	Year	Component	Procedures				Data source			
			FD	FDE	FI	FP	Visual	Acoustic	Vibration	Others
[136]	2013	Rotor		X		X			X	
[16]	2013	Bearing	X					X		
[71]	2014	Bearing, Shaft, Rotor	X				X		X	
[91]	2014	Bearing, Gear, Rotor	X			X(c)			X	
[89]	2014	Belt-pulley	X		X				X	
[138]	2015	Rotor	X		X				X	
[72]	2015	Bearing, Shaft	X						X	
[24]	2016	Bearing, Gear	X					X	X	
[137]	2016	Rotor		X		X(c)			X	
[7]	2016	Bearing, Stator, Rotor	X		X					ES
[41]	2017	Bearing	X			X(c)			X	
[90]	2017	Machine Faults	X						X	
[86]	2017	Shaft, Rotor	X						X	
[140]	2017	Rotor		X		X(c)				ES
[135]	2018	Rotor	X				X			
[98]	2018	Bearing, Gear	X				X			
[21]	2018	Bearing, Gear, Shaft	X						X	
[100]	2018	Bearing, Rotor	X						X	
[11]	2019	Bearing	X				X			
[23]	2019	Bearing	X					X		
[127]	2019	Bearing, Gear	X			X(c)			X	
[133]	2019	Rotor	X				X			
[134]	2019	Rotor		X						ES
[22]	2019	Bearing, Rotor	X						X	
[46]	2019	Shaft		X			X			
[40]	2019	Bearing, Gear, Rotor	X						X	
[109]	2020	Bearing	X		X		X			
[111]	2020	Bearing	X			X(c)			X	
[115]	2020	Bearing, Rotor	X				X			
[8]	2020	Gear	X						X	
[79]	2019	Rotor	X				X		X	
[142]	2020	Journal Bearings		X					X	
[107]	2021	Bearing, Gear	X			X(c)			X	
[13]	2021	Bearing, Gear		X						SCADA
[27]	2021	Gear	X						X	TQ, PR
[12]	2021	Rotor	X			X(c)	X			
[132]	2021	Bearing, Rotor	X						X	
[85]	2021	Gear		X					X	
[104]	2022	Bearing	X				X		X	
[101]	2022	Bearing, Gear		X					X	
[75]	2022	Bearing, Gear	X						X	
[84]	2022	Gear	X						X	TM
[80]	2022	Rotor		X			X			
[15]	2022	Bearing, Turbine blades	X	X					X	
[141]	2022	Rotor		X		X				ES
[11]	2023	Bearing, Rotor	X					X	X	TM
[139]	2023	Rotor		X		X(c)			X	
[125]	2023	Bearing	X			X(c)	X			
[29]	2023	Bearing				X	X		X	SCADA

Kang et al. [71] used two-dimensional gray-level images for the FD of the bearing, shaft, and rotors of rotating machines. For this purpose, they employed wavelet transform (WT) considering Shannon wavelets for multiresolution analysis of the vibration signals. They considered angular misalignment, broken rotor bar fault, rotor unbalance fault, bearing outer race fault, bowed shaft, and bowed rotor faults to assess their proposed approach. Li et al. [24] considered acoustic emission and vibration signals with several ML algorithms for the FD of bearing and gear faults. They used a benchmark dataset where gear wore tooth, chaffing tooth, pitting tooth, chipped tooth, root crack tooth, bearing inner race fault, bearing outer race fault, bearing ball fault, and bearing house eccentricity exist. Zolfaghari et al. [140] performed the FDE procedure of rotors in a rotating machine regarding broken rotor bar fault cases. They used the current signal acquired from the stator to represent the healthy and faulty components. Chen et al. [27] used vibration, torque, and pressure data for FD of a planetary gearbox existed in a wind turbine drivetrain diagnosis simulator. They considered five conditions of the planetary gearbox including healthy gear, surface worn fault, missing tooth, chipped tooth, and cracked tooth. Zhao et al. [29] conducted an FP procedure to predict the RUL of a bearing component of the direct-drive wind turbine. For this purpose, they considered a digital-twin-based method where environmental parameters and operational parameters are given as input to constitute a virtual output in terms of vibration signals. Afterwards, they used actual vibration data obtained from the main bearing of a wind turbine. Both vibration data are then processed to build an intelligent FP model.

### 6.2 Feature extraction

IFDP models require the specific features of the given data to constitute meaningful relationships to give accurate results. There are various feature extraction methods used in the IFDP of rotating machines. *Time-domain analysis* is used to express the behavior of the signals acquired from the machine in the time domain. Some time-domain features are peak values, root means square values, and crest factors. *Frequency-domain analysis* transforms the acquired raw data into the frequency domain by considering specific techniques such as Fast Fourier Transform, Wavelet Transform, Empirical Mode Decomposition, and Singular Value Decomposition. Some frequency-domain features are spectral energy, bandwidth, dominant frequency, etc. Statistical feature extraction comprises the evaluation of the statistical metrics including mean, median, nth percentile, kurtosis, skewness, and standard deviation from the raw data. *Signal processing methods* process the given data by filtering, normalizing, standardizing, or smoothing to find suitable features. Apart from those manual techniques, machine learning algorithms are also used to find the patterns in the raw data and find the relevant features automatically.

**Fig. 15:**
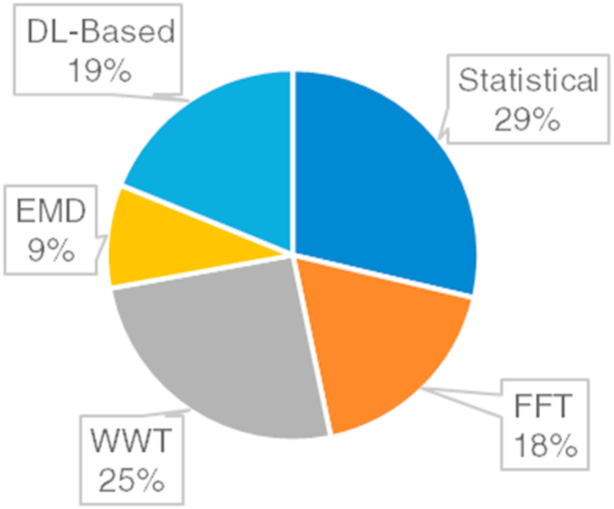
Feature extraction techniques used for IFDP of rotating machines.

The acquired data sources are subjected to manual or automatic feature extraction procedures for IFDP procedures. The most feature extraction techniques regarding FD, FDE, FI, or FP procedures of rotating machine components are statistical feature extraction (STFE) [10,27,29,101], Fast Fourier Transform (FFT) [8,33,34,109], Wavelet Transform (WWT) [20,73,78,134], and EMD [21,41,52]. There are also some methodologies derived from those techniques including Discrete Fourier Transform (DFT) [95], Short Time Fourier Transform (STFT) [36], Wavelet Packet Transform (WPT) [37,83], Continuous Wavelet Transform (CWT) [36,38], Discrete Wavelet Transform (DWT) [76,122], and Ensemble Empirical Mode Decomposition (EEMD) [112,121]. There are also other methods such as Singular Value Decomposition (SVD) [42,82,121], Similarity-Based Modelling (SBM) [100], and Hilbert Transform [35,42] that are all considered manual feature extraction techniques. On the other hand, developed ML models such as Convolutional Neural Networks (CNN) [106,107], Generative Adversarial Networks (GAN) [108], Long-Short Term Memory (LSTM) [83,107,130], and Autoencoders (AE) [87,105] are used for automatic feature extraction procedures. Considering the popular feature extraction strategies used for IFDP of rotating machines, it is seen from Fig. 15 that the statistical features of raw signals or processed signals are the most preferred method followed by WT-based techniques, deep learning-based (DL-based) methods, FFT-based techniques, and EMD-based approaches, respectively.

Table 6 show the studies related to IFDP of rotating machines considering the employed feature extraction and data types.

**Table 6:** Intelligent fault diagnosis and prognosis studies and feature extraction methods.

Studies	Year	Component	Data Types	Feature extraction				
				STFE	FFT	WWT	EMD	Other
[16]	2013	Bearing	Acoustic	X				
[6]	2013	Bearing, Rotor	Voltage, Current	X	X			X
[71]	2014	Bearing, Shaft, Rotor	Visual, Vibration			X		X
[92]	2015	Bearing, Gear	Vibration	X	X			
[93]	2015	Bearing, Gear	Vibration	X		X		
[122]	2015	Bearing	Vibration			X		X
[123]	2015	Bearing	Vibration			X		X
[72]	2015	Bearing, Shaft	Vibration					X
[24]	2016	Bearing, Gear	Acoustic, Vibration			X		
[94]	2016	Bearing, Rotor	Vibration	X	X			
[31]	2016	Bearing	Vibration	X			X	
[87]	2016	Bearing, Stator, Rotor	Vibration					X
[41]	2017	Bearing	Vibration	X		X		X
[95]	2017	Bearing, Gear	Vibration					X
[119]	2017	Bearing	Vibration	X	X		X	
[121]	2017	Bearing	Vibration				X	X
[73]	2018	Bearing	Vibration	X		X	X	
[97]	2018	Bearing, Gear	EMG	X		X		
[21]	2018	Bearing, Gear, Shaft	Vibration					X
[99]	2018	Bearing, Gear	Vibration			X	X	
[48]	2018	Bearing	Vibration	X				
[116]	2019	Bearing	Visual			X		
[23]	2019	Bearing	Acoustic			X		
[126]	2019	Bearing	Visual		X			
[44]	2019	Bearing	Vibration					X
[40]	2019	Bearing, Gear, Rotor	Vibration	X			X	X
[108]	2020	Bearing	Vibration					X
[50]	2020	Bearing	Vibration					X
[109]	2020	Bearing	Visual		X	X		X
[47]	2020	Bearing	Visual, Current					
[110]	2020	Bearing	Vibration		X			
[111]	2020	Bearing	Vibration		X			X
[112]	2020	Bearing	Vibration	X			X	
[115]	2020	Bearing, Rotor	Visual					X
[37]	2021	Bearing	Vibration			X		
[14]	2021	Bearing	Vibration		X			X
[32]	2021	Bearing	Visual	X				
[35]	2021	Bearing	Vibration		X			X
[107]	2021	Bearing, Gear	Vibration					X
[13]	2021	Bearing, Gear	SCADA					X
[132]	2021	Bearing, Rotor	Vibration		X			
[34]	2021	Bearing, Rotor	Visual		X			
[52]	2022	Bearing	Vibration					X
[104]	2022	Bearing	Visual, Vibration			X	X	
[10]	2022	Bearing	Vibration	X		X	X	
[46]	2022	Bearing	Vibration					X
[101]	2022	Bearing, Gear	Vibration	X				X
[75]	2022	Bearing, Gear	Vibration					X
[105]	2022	Bearing	Vibration					X
[15]	2022	Bearing, Turbine blades	Vibration					X
[11]	2023	Bearing, Rotor	Acoustic, Vibration, Tachometer	X		X		
[125]	2023	Bearing	Visual					X
[29]	2023	Bearing	Visual, Vibration, SCADA	X			X	X

### 6.3 Fusion techniques

There are several fusion methods in machine learning models to develop effective intelligent techniques. Fusion techniques are referred to as combining data or related information from different sources at various levels. By using fusion techniques, it is possible to obtain ML models that give more accurate and robust decisions.

In general, the fusion procedures are constituted from three levels. *Data fusion* is related to combining data from different sources to improve the representability of an intelligent model. For instance, data fusion may be effective when combining data collected from the same data sources which are placed in different locations of machinery [148,149]. A fault's signature may appear differently in those sources. However, that difference may result in a positive impact on the performance of the intelligent model. Based on this example, multi-modal sensor fusion or multi-location sensor fusion methods are effective approaches in data fusion. *Multi-modal sensor fusion* [148] comprises combining data acquired from distinct sensors (e.g., accelerometer + microphone, tachometer + thermal cameral + acoustic emission sensor, etc.) that measure different modalities or any other different physical data. By doing so, the limitations of each sensor may be mitigated. *Multi-location sensor fusion* [149] is the integration of the data collected from the same or different sensors that are placed in different locations. It is mainly considered to improve the accuracy and reliability of an intelligent model. Multi-location sensor fusion may require additional techniques including Kalman Filters, Bayesian Networks, or different regression methods. *Feature fusion* denotes the integration of the feature sets extracted from the same or different sensors [150]. For instance, a bearing fault may be found by using the feature sets obtained from a thermal camera and accelerometer. *Decision fusion* is related to giving a final decision by combining the decisions made by different intelligent methods based on different data sources and feature sets [151].

Fusion techniques are useful to solve complex problems, especially where the error tolerance is required to be very small. By using fusion techniques, improved accuracy, robustness, and reliability may be provided.

Regarding the IFDP of rotating machines, there are only a few studies that investigated the effectiveness of the data fusion techniques regarding multi-modal sensor fusion, feature fusion, and decision fusion [9,11,24,27,29]. Most IFDP approaches seek the use of a single data source obtained from a single sensor or multi-location sensor fusion approach where data is acquired from the same type of sensors placed in different locations on the components of the rotating machine.

### 6.4 Machine learning and data mining

**Fig. 16:**
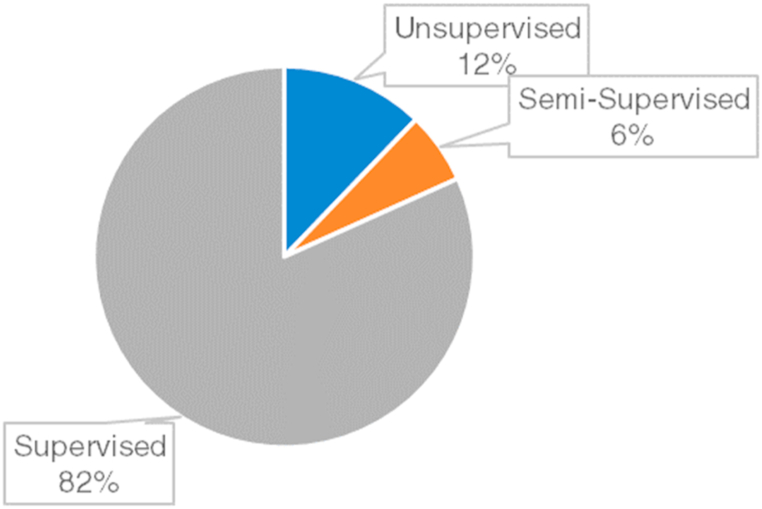
The learning approaches considered for IFDP of rotating machines.

ML can be categorized as supervised learning, unsupervised learning, and semi-supervised learning. ML models are used in a vast range of areas including image recognition [1,2], speech recognition [4], recommendation systems [152], autonomous driving systems [153,154], etc. Supervised learning is based on learning the relationships between the input and output. The performance of the model is assessed by testing it with unseen data. The inputs may be categorical, numeric, ordered values, statistical values, time-series values, or matrices. The outputs may be categorical data (classification) or continuous data (regression) [[155], [156], [157]]. In other words, a supervised learning-based ML algorithm can identify the fault type of a faulty machine (classification) or aim to predict a continuously changing fault severity value (regression). Supervised learning-based algorithms may be linear (Logistic Regression, Linear Discriminant Analysis) or non-linear (Naive Bayes, Support Vector Machines, Non-Linear Logistic Regression). In multi-dimensional heterogeneous datasets, there are mostly non-linear relationships among the data. It is necessary to use suitable models that can express these relationships effectively. In semi-supervised learning, labeled and unlabeled data are used together to train the ML model. In this kind of learning strategy, the majority of the training data is unlabeled. Semi-supervised learning is generally used in problems where lots of data exist and labeling the data is difficult or costly [[155], [156], [157]]. Unsupervised learning is another learning strategy where labeling is based on extracting patterns and relationships among the unlabeled input data [[155], [156], [157]]. Some unsupervised learning techniques are Association Rule Mining, Clustering, and Anomaly Detection. In an ARM task, the co-occurrences of the inputs are calculated. On the other hand, clustering is about aggregation based on the patterns between the data. Anomaly detection clusters the data by finding and discarding the outliers. As seen in Fig. 16, the majority of the studies considered supervised learning approaches followed by unsupervised learning and semi-supervised learning.

**Fig. 17:**
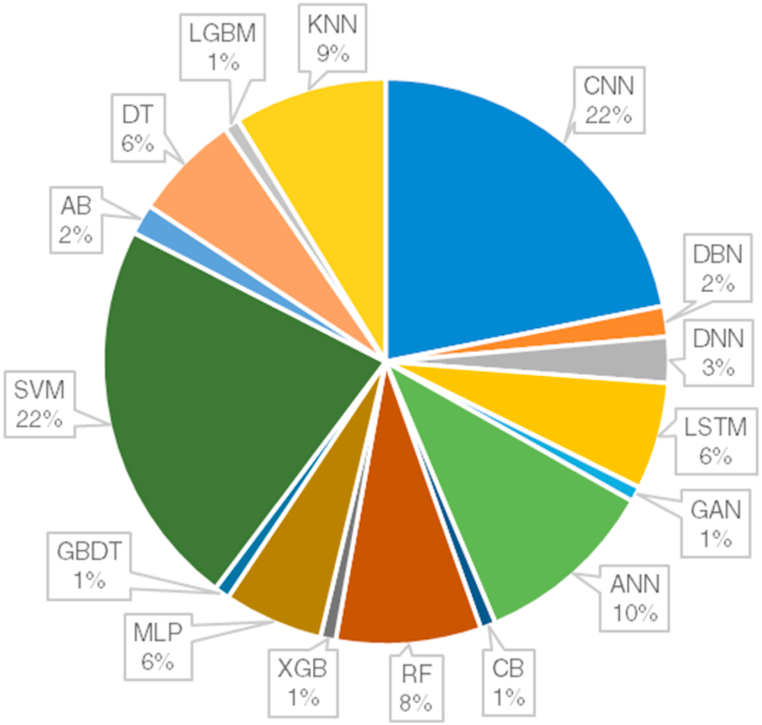
The usage distribution of the ML algorithms in the field of IFDP of rotating machines.

In the field of rotating machines, various ML algorithms are used or improved to propose an effective IFDP method. Table 7 shows the studies related to the IFDP of rotating machines regarding the considered components and ML algorithms. It is seen from Table 7 that the use of popular single learners such as Support Vector Machines (SVM) [24,41,87,88,117], K-Nearest Neighbors (KNN) [44,90,96,115], and Decision Tree (DT) [16,17,74] are widely used for IFDP of bearing [16,41,78,120], gear [8,27,83,130], rotor [26,134,137], shaft [46,71], and stator [7,87]. On the other hand, ensemble learning approaches such as Random Forest (RF) [81,94,97], AdaBoost (AB) [8,11,113], Gradient Boosting Decision Tree (GBDT) [14], Light Gradient Boosting Machine (LGBM) [12], eXtreme Gradient Boosting (XGB) [14], and CatBoost (CB) [52] are rarely used when compared with single learners, deep learning approaches, and neural networks. These approaches are considered for IFDP of bearing [97,100,116], gear [43,81,97], and rotor [26,100]. Neural networks such as Artificial Neural Networks (ANN) [7,30,39,122,140] and Multilayer Perceptrons (MLP) [8,25,47,95] are extensively used for IFDP of bearing [39,74,122], gear [8,27,83] rotor [25,86,134,140], stator [25], shafts [46,86], and other components [89] of rotating machines. Deep learning algorithms are increasingly considered the IFDP of rotating machines. Especially the use and improvement of Convolutional Neural Networks (CNN) [29,92,119,125,126,135,139] have drawn the attention of many researchers as seen in Table 7. Deep learning approaches such as Deep Belief Neural Networks (DNN) [15,114,127], Deep Neural Networks (DNN) [14,15,87,114,120], Long-Short Term Memory (LSTM) [29,84,101,107,132], Generative Adversarial Network (GAN) [108], Autoencoder (AE) [34,105,111,114], Domain Adaptive Neural Networks (DANN) [45,75,125,139] are considered for bearing [73,75,87,119,120,125], gear [43,75], rotor [87,133,135,139], stator [87], shaft [46], and other components [15,28] regarding FD, FDE, FI, and FP procedures. As seen from Fig. 17, SVM and CNN-based ML algorithms are the most used techniques for the IFDP of rotating machines. On the other hand, ensemble learning methods are the least considered algorithms in this field.

**Table 7:** Intelligent fault diagnosis and prognosis studies and used machine learning algorithms.

Studies	Year	Component	Algorithm																
			CNN	DBN	DNN	LSTM	GAN	ANN	MLP	CB	XGB	LGBM	GBDT	RF	AB	DT	SVM	KNN	Others
[16]	2013	Bearing														X			
[22]	2013	Stator, Rotor							X										X
[120]	2014	Bearing						X											X
[88]	2015	Bearing, Gear	X														X		
[118]	2015	Bearing						X	X										X
[21]	2016	Bearing, Gear												X			X	X	X
[90]	2016	Bearing, Rotor												X			X		X
[17]	2016	Gear														X			X
[83]	2016	Bearing, Stator, Rotor			X			X									X		X
[91]	2017	Bearing, Gear						X	X								X		X
[92]	2017	Bearing, Gear	X														X	X	
[40]	2017	Gear	X					X						X			X		
[82]	2017	Shaft, Rotor						X									X	X	X
[116]	2017	Bearing			X												X	X	
[136]	2017	Rotor						X	X										
[69]	2018	Bearing	X			X													
[70]	2018	Bearing, Gear						X								X			
[131]	2018	Rotor	X														X		X
[93]	2018	Bearing, Gear												X				X	
[45]	2018	Bearing														X	X		X
[41]	2019	Bearing	X					X									X	X	
[123]	2019	Bearing, Gear	X	X															
[46]	2019	Bearing, Rotor	X											X			X		
[126]	2019	Gear	X			X		X									X		
[130]	2019	Rotor	X						X								X		
[43]	2019	Shaft	X						X								X		
[104]	2020	Bearing	X				X												X
[44]	2020	Bearing	X						X								X	X	
[106]	2020	Bearing	X																X
[125]	2020	Bearing, Gear	X			X								X			X		
[109]	2020	Bearing, Gear	X											X	X	X	X		
[110]	2020	Bearing, Rotor	X	X	X														X
[111]	2020	Bearing, Rotor	X													X	X	X	
[8]	2020	Gear				X			X					X	X	X	X	X	X
[9]	2020	Gear	X													X	X	X	X
[14]	2021	Bearing	X		X						X		X	X			X		
[29]	2021	Bearing	X					X											
[32]	2021	Bearing	X											X			X		X
[103]	2021	Bearing, Gear	X			X													
[13]	2021	Bearing, Gear							X							X	X	X	
[24]	2021	Gear						X						X			X	X	X
[124]	2021	Gear															X		X
[12]	2021	Rotor	X									X					X	X	X
[128]	2021	Bearing, Rotor				X													X
[49]	2022	Bearing				X				X									
[10]	2022	Bearing												X		X	X		X
[42]	2022	Bearing	X																X
[97]	2022	Bearing, Gear				X											X		
[80]	2022	Gear	X			X													
[99]	2022	Gear				X										X	X		X
[76]	2022	Rotor	X																
[11]	2023	Bearing, Rotor						X						X	X				
[135]	2023	Rotor	X																X
[121]	2023	Bearing	X																X
[26]	2023	Bearing	X			X													X

### 6.5 Types of rotating machines and real-world settings

There are numerous types of rotating machines developed for a specific purpose used in the industry. Each type has different challenges in fault analysis. For instance, the operational conditions of a centrifugal pump may significantly alter depending on the type of fluid, fluid pressure, temperature, and variable system needs. All of these parameters make challenging the identification of faulty or even healthy pumps. Another example may be given for gas and steam turbines. These machines operate at very high temperatures which makes collecting and processing data by a mounted sensor compelling. Regarding those machines, sensor placement, effective data collection, and effective data processing may be considered key difficulties. As one of the most common rotating machine types, wind turbines bring specific challenges in intelligent fault analysis. The collected data is highly affected by external noise, aerodynamic forces, and environmental conditions. For instance, vibration and acoustic data are remarkably affected by the excessive noise generated by the turbine itself and the aerodynamic forces developed by the wind. In such machines, multi-modal fault diagnosis may be more beneficial to reduce such drawbacks. Similar challenges are observed for each rotating machine and therefore, it is essential to understand the machine-specific difficulties when collecting and processing data and constituting an IFDP model.

As presented in the former sections, numerous studies are conducted in the field of fault analysis of rotating machines. A majority of those studies considered benchmark datasets collected from hand-crafted laboratory test beds having a motor that drives a shaft connected to a set of gears and/or supported with bearings. Such test beds are also denoted as machine fault simulators. Some other built an experimental setup to represent the rotating machines or directly consider the real-world rotating machines, including wind turbines [13,99,125,130,[158], [159], [160]], rotor test rigs of aircraft [8], compressors [[161], [162], [163]], gas turbines [164,165], steam turbines [166,167], pumps [[168], [169], [170]], train bogie [171] and other rotating machine-included systems such as unmanned underwater or aerial vehicles [8,172] for machine learning-based fault analysis. Some recent works regarding the fault diagnosis of the rotating machines mentioned above are presented as follows.

Han and Li proposed an out-of-distribution assisted trustworthy machinery fault diagnosis considering bearing faults of a wind turbine and gear fault of a single-stage planetary gearbox. They integrated deep ensemble neural networks to constitute an ensemble fault diagnosis structure. The ensemble was established by five individual deep base learners including a three-convolutional layered CNN, four-convolutional layered CNN, five-convolutional layered CNN, a gradient-skipping ResNet architecture, and an interception block built by GoogleNet. They compared the results of the deep ensemble learning with each individual deep base learner. They concluded that the developed deep ensemble learning method was superior to others and gave promising results in handling the problem of unseen faults [158].

Zhang et al. employed a convolutional deep belief network-based fault diagnosis approach for reciprocating compressors. They developed an auto-denoising network in which the signal is denoised and the most representative features are extracted. To evaluate the probability of fault occurrence, they adopted multiple Gaussian process classifiers. Following the combination of those properties considering an optimized weight, the fault type is identified. They concluded that their proposed approach effectively diagnosed the reciprocating compressor faults with accuracy rates of up to 91.89% [161]. Hasan et al. presented a grayscale-converted scalogram-based adaptive deep convolutional neural network (ADCNN) for fault diagnosis of centrifugal pumps. For this purpose, they collected the vibration signals and decompose them by using Continuous Wavelet Transform to obtain scalograms. Afterwards, they converted the scalograms into grayscale images to be fed as input to ADCNN. They compared their proposed approach with different fault diagnosis models such as CNN, FFT + CNN, and Statistical Feature Extraction + kNN. They concluded that the developed approach outperformed these models by an improvement in accuracy of up to 15.6% [168]. Manikandan and Duraivelu developed a deep CNN-based approach for fault diagnosis of the impeller and mechanical seal in industrial mono-block centrifugal pumps. For this purpose, they converted the collected vibration signals into 2D images. They trained and test the deep CNN classifier using such image data. They concluded that their proposed approach identified the faults with an accuracy value of 99.07% [170]. Ding et al. proposed a multiscale lightweight network with adaptive pruning for fault diagnosis of train bogie bearings. They constituted weight-sharing multiscale convolutions to obtain the multi-time scale features of vibration signals. Afterwards, they structured the inverse separable convolution blocks to obtain highly representative features. Besides, they implemented an adaptive pruning technique to eliminate redundant network structures during training. They concluded that the developed technique is superior to other recent methods in fault diagnosis of lightweight bearings working under variable conditions [171].

Some of those studies indicated that their proposed approach is based on real-time since the data that they used are acquired from one or multiple experimental setups that are given above. It is controversial to consider such approaches as a real-time-based IFDP method since gathering the data from a rotating machine in real-time does not precisely mean that the constituted model is going to operate in real time with the same performance. Besides, in real-time, the IFDP model may completely fail in distinguishing the healthy machine from the damaged one or identifying the correct fault type and/or fault severity due to improper algorithm and/or parameter selection or the differences in the environmental/operational conditions of the same machine used to train the model. Although the hand-crafted fault simulators or rotating machines that are developed in laboratories may mimic real-world operating machines, they still remain as developed or tested in a laboratory environment. All such laboratory equipment are denoted as idealized since they do not completely represent real-world settings such as environmental parameters (i.e., temperature, humidity, pressure, and ambient noise) and operation conditions (i.e., motor speed, load, voltage, disruptive or coupled vibrations). In addition, the fault types existing in those laboratory setups are artificial and therefore, do not depict the natural forming of the faults. This may cause deceptive or ineffective outcomes since it is essential to detect, localize, and identify the fault in its early-stage to plan or execute the maintenance procedure.

Some studies in the literature comprise the real-time assessment of their proposed approach by developing an interface where a simulation including data acquisition, data processing, and fault analysis procedures are conducted sequentially regarding the same machine in real-time after training the model [22,38,[172], [173], [174]].

Lee et al. used CNN for fault diagnosis of induction motors in real time. They considered rotor and bearing faults (punctured rotor, worn bearing) for the fault detection procedure. They employed a simple pipeline where the data is collected via a vibration sensor and sent to the build model from a data acquisition device. They constituted an interface in LabVIEW software in which all the steps of the fault diagnosis can be monitored easily. They achieved an overall accuracy of 98.66% regarding the identification of the normal state and all faulty conditions of the machine [22]. Guo et al. proposed a multi-task CNN with information fusion for bearing fault diagnosis. They included an online data processing step in their approach where the data is given to multi-task CNN as input for fault diagnosis. They tested their proposed approach considering two benchmark datasets, namely Case Western Reserve University Data (CWRU) and Machinery Fault Simulator – Rotor Dynamics Simulator Bearing Data (MFS-RDS) where bearing faults exist. They achieved an average accuracy of 95.04% regarding fault identification and localization in real-time [38]. Bagci Das and Birant used the combination of genetic algorithm and ensemble learning (GASEL) for fault detection of autonomous underwater vehicles (AUV). The vehicle includes propeller damages with two severities, load-increase fault, and sensor faults. They assessed the performance of their proposed approach by employing Flask environment in Phyton and Postman API to receive the raw data and send it to the fault diagnosis module. They achieved an average accuracy of 99.33% including the prediction of the healthy state and all faulty conditions of the vehicle [172]. Zhao et al. combined transfer learning and embedded convolutional LSTM with digital twin technology for bearing life prediction in wind turbines. For this purpose, they generated the digital twin of the vibrational behavior of the main bearing of the wind turbine in the ANSYS environment. They tested their proposed approach regarding two benchmark datasets, namely IEEE Prognostics Health Management (PHM) Data Challenge and XJTU-SY. Afterwards, they conducted a second experiment on a real-world 1.5 MW wind turbine in Inner Mongolia. Regarding the real-world test results, they predicted the life of the bearing several months earlier than its actual lifespan [173]. Syafrudin et al. proposed a Density-Based Spatial Clustering (DBSCAN) with Noise-based outlier detection and RF for real-time fault detection in an automotive assembly line process. For this purpose, they utilized Apache Kafka, Apache Storm, and Mongo DB to handle the real-time procedures. They achieve a perfect score regarding fault detection when using DBSCAN with RF [174].

Such studies given above provide promising outcomes regarding the performance of their proposed IFDP methods when they are operated in real-time conditions. On the other hand, they still do not satisfy the real-world settings thoroughly since the IFDP approaches developed in those studies are constituted in a conservative environment that mostly neglects the environmental and operational variable parameters.

It is seen from the literature survey that the implementation of IFDP models in real-world settings is rarely explored. The development of the IFDP models that satisfies the uncertainties due to environmental and operational parameters is ultimately required to constitute robustness and reliance in the field of intelligent fault analysis of rotating machines. This may require time since it takes time for fault development in normal operation. Such time consumption may be reduced by improper use of the machine. However, doing so may result in the complete failure of the machine and may even cause undesired costly consequences. Regarding real-world applications, a semi-supervised or unsupervised technique may be more beneficial since gathering the faulty data to train an IFDP model is challenging as there are numerous fault types that show up in certain circumstances and requires time as mentioned above. However, using semi-supervised or unsupervised methods may cause a struggle in the identification of fault type since a semi-supervised or unsupervised IFDP method may mispredict the fault type due to the similar or distinctive characteristics that show up in the vitals of a machine. Hence, a blend of supervised and semi-supervised/unsupervised methods may give more accurate and reliable results in real-world applications. Such a combination may be denoted as another important feature direction. Finally, it is favorable to use an IFDP model that effectively operates in different machines in the real world. For instance, it is preferred to constitute an IFDP model that successfully performs fault analysis in gearboxes both used for wind turbines and for industrial reduction gearboxes. For such purposes, numerous studies are focused on transfer learning-based approaches [75,103,118,139,165] to achieve an IFDP model as generalizable as possible. Although generalizability is an important aspect of IFDP procedures, it is more critical to focus on a specific rotating machine in real-world settings since it is possible to develop distinct IFDP models for each machine in a factory that are in communication with each other in a harmony with the concept of Industry 4.0. On the other hand, from the perspective of Industry 4.0, it is a must to use a reliable and robust IFDP approach that gives accurate, meaningful, and explainable results in real-time.

## 7 Summary and recent challenges

This review encompasses the literature related to IFDP of rotating machines regarding various aspects including machine components, fault types, fault existence, fault analysis strategy, data sources, data fusion, feature extraction, and machine learning technique. It is seen that the most examined component is the bearing followed by the gear, rotor, shaft, stator, and other parts (RQ1).

Regarding the fault types, bearing faults are investigated the most but are limited to inner race, outer race, cage, and ball faults. Following the bearing faults, gear and rotor faults are commonly considered. For gear faults, crack, wear, chipped tooth, broken tooth, missing tooth, and pitting faults are widely remarked. For rotor faults, broken rotor, misalignment, unbalance, and rub-impact faults are extensively considered. Stator, shaft, and other faults are rarely taken into account and the IFDP approaches regarding those parts are scarce (RQ2).

To constitute an IFDP approach various data sources including, vibration, acoustic, thermal, pressure, torque, current, and voltage data are considered in the form of 1-D signal or visual data. Among these data sources, vibration is the most utilized one followed by acoustic data (RQ3).

It is possible to improve the performance and reliability of an IFDP model by employing data fusion techniques. The data fusion procedure is conducted at various levels in intelligent fault analysis of rotating machines. It is seen that the fusion process is usually performed at the data level, including multi-modal sensor fusion and multi-location sensor fusion in the literature related to the IFDP of rotating machines. Apart from those techniques, feature level fusion and decision level fusion are also considered in the literature (RQ4).

Regarding the feature extraction techniques, hand-crafted techniques such as statistical features, FFT-based features (STFT and DFT), WWT-based features (DWT, CWT, and WPT), and EMD-based features (EEMD, CEMD, CEEMDAN) are considered based on the data source. In numerous studies, raw signals are directly used or processed to obtain visual or signal data to be used especially for deep learning-based techniques. These techniques may include feature extraction layers that obtain the characteristic properties of a given data automatically (RQ5).

The machine learning approaches used for the IFDP of rotating machines comprise supervised, unsupervised, and semi-supervised learning. In general, supervised learning approaches are considered for FD, FDE, FI, and FP of rotating machines. All fault analysis strategies are considered mostly as classification problems. On the other hand, it is more meaningful to pursue a regression-based approach for FP procedures since fault severity is a continuous parameter. Besides, FP is widely conducted to help or assess the RUL of a component. Hence, adopting a classification-based approach regarding FP only provides the capability of a model in catching fault severity values at a certain level and therefore, does not present the trend of component condition that helps to find its RUL (RQ6).

A variety of machine learning techniques are used or improved to develop effective IFDP approaches regarding rotating machines. In the last decade, it is seen that Support Vector Machines (SVM) and Convolutional Neural Networks (CNN) are the most used machine learning techniques in the field of IFDP of rotating machines. In addition, Neural Network-based approaches and K-Neareast Neighbors are commonly used in this field (RQ7).

The developed IFDP approaches are assessed mostly by overall model accuracy regarding the FD procedures. On the other hand, based on the considered machine learning approach (classification, regression, or clustering), precision, recall, confusion matrix, F1 score, AUC, ROC, mean squared error, root mean squared error, mean absolute error, and time consumption are considered (RQ8).

Introducing artificial intelligence to fault analysis of a machine has a significant impact on the maintenance strategy. Since the IFDP procedures involve the condition monitoring and assessment of the RUL of a component or a machine the best strategies can be predictive maintenance (PdM) and condition-based maintenance (CBM) (RQ9).

Although fault analysis is a helpful task, it has some challenges that should be overcome. The main challenges are given below (RQ10).

• Data collection: A very common challenge is the collection of relevant and accurate fault data because of its strong impact on the performance of machine learning methods. The rotating machines have numerous operational indicators including vibration, speed, temperature, pressure, current and voltage data that may be useful for intelligent fault analysis. On the other hand, some IFDP techniques such as deep learning-based approaches usually require higher amounts of data than other ML algorithms regardless of the data type. To satisfy such a limitation, it is needed to acquire such an amount of data for both healthy and faulty machines. It is challenging to gather data from a faulty machine in real-world settings since a machine operates mostly in healthy conditions. The level of such a challenge increased even higher when a data-hunger ML method is adopted for fault analysis.
• Big data: With the recent development of smart manufacturing and the Internet of Things, the amount of collected fault-related data has grown in an exponential manner and has high-dimensional characteristics. Besides, each machine condition poses uniqueness in the collected data that increase the complexity of the problem. It is challenging to overcome such an issue with fewer data since IFDP methods have limited capabilities to conduct the learning process grounded on real-world physics. Besides, they do not comprehend the ties between cause and effect and therefore, do not come up with a conclusion based on such a relationship. Hence, the requirement for big data becomes inevitable, especially for more complex problems such as observing multiple kinds of faults at the same time. Analyzing large fault data is also a challenging task since it requires the use of powerful big data analytical techniques (i.e., feature dimensionality reduction methods), specialized algorithms and methodologies, and a vast amount of storage and computing resources.
• Imbalanced data: The fault-related data collected from rotating machinery in a real-world industrial system is highly imbalanced since there are few machine failures relative to the number of normal conditions. The data imbalance may result in fallacious results as it is possible that a developed IFDP method may predict all instances as healthy by discarding all faulty cases. This situation may result in high accuracy, but poor precision and recall. Hence, the data imbalance constitutes another challenge to achieving effective standard machine-learning solutions for fault analysis.
• Data pre-processing: Data pre-processing is a significant challenge in the field of IFDP of rotating machines since each kind of data has a different impact on the model performance. It is required to obtain the most representative features to feed the ML model to achieve successful results. However, the selection of the signal or image processing method, tuning its parameters, and extracting the right features requires tedious work and expertise. For instance, regarding 1D time series signals, using Fast Fourier Transform or Wavelet Transform generally constitute differences in model performance. Similarly, parameter tuning in signals processing (e.g., sliding window time, overlap ratio, decomposition level, wavelet type, etc.) affected the model performance considerably as they unearth or hide the fault-specific features that existed in the data.
• Dynamic system: Due to the fast-changing and dynamic Industry 4.0 environment, the machine learning system should be capable to learn and flexible under changing conditions. The machine learning model should be continuously updated by the newly collected data. In addition, it is challenging for an IFDP model under variable physical conditions including operational and environmental parameters such as temperature, humidity, pressure, ambient noise, coupled vibrations, etc. An effective IFDP model or models have to recognize the changes in such parameters and make predictions accordingly. In general, IFDP models are used to solve a particular problem under confined conditions. Therefore, their model performance would be significantly affected by changes in physical conditions. Such a limitation constitutes an extreme challenge regarding the development and employment of IFDP models in real-world settings.
• Algorithm selection: Another significant difficulty is the selection of the machine learning technique. Each machine learning study is unique in its context since the data availability affects the algorithm's performance. Thus, the best machine learning method that is capable to solve the target fault analysis problem is not clear. Selecting the appropriate learning strategy stands as another challenge. Although IFDP techniques have a high capability in distinguishing data with different labels, it may not be useful to employ supervised learning techniques in cases where an excessive amount of diversity exists in data. In such conditions, it is more convenient to pursue a fault detection strategy rather than fault identification. Supervised algorithms require labeled data for learning. On the other hand, it is difficult to constitute a dataset including all kinds of faults to train the supervised model due to the possibility to obtain faulty data and the issues related to big data as mentioned above. In addition, the suitability of the chosen IFDP method may change for different fault cases. It is expected that the researchers will be aware of potential ML algorithms suitable for a given problem/data and will compare their performances based on various metrics.
• Optimal parameter selection: The performance of each algorithm on fault detection depends on the parameter settings. The optimal parameter settings can be determined based on a lot of experimental work, which is computationally expensive. It is challenging to assess the optimal parameters since each fault type has a unique characteristic impact on the data which may require a more complex or more simple algorithm. Hence, it may be necessary to conduct a fault-specific parameter tuning of the selected IFDP approach.
• Evaluation: The assessment of the machine learning model in terms of understanding and interpreting the results obtained in fault analysis is one of the most challenging processes. The question arises as to how to measure the performance and assess the validity of the results. It is also necessary to prevent the underfitting and overfitting of the models. Hence, it is needed to provide a balanced and satisfying amount of data for training since an IFDP approach may be prone to memorize the characteristics of the data instead of learning may result in false predictions in the test set (overfitting) or may not learn anything due to insufficient amount of data (underfitting). Another challenge is testing a proposed IFDP approach with unseen data that have different characteristics from the training data in one way or another. In addition, a proposed method is desired to be tested under real-time and real-world conditions. Although it is possible to test a model in real-time by using distinctive data, assessing the model in real-world settings is extremely challenging and time-consuming since the fault growth procedure may take a long time and the consequences of a fault may result in catastrophic failure of the machinery if the fault is not detected by the model. Another issue regarding the evaluation of a proposed IFDP approach is dealing with unbalanced data. In the field of fault diagnosis of rotating machinery or any kind of machine, the amount of healthy machine data is abundant since the machine operates normally in most cases. On the other hand, collecting faulty data is challenging and even if it is collected, it will not be as plenty as that of the healthy data because the machine eventually has to stop since operating a machine in a faulty condition poses a significant danger. Unbalanced data may result in fallacious results such that a developed IFDP model may be successful in predicting the healthy condition while it may completely fail to assess the faulty machine. Even in such a scenario, the overall accuracy of the IFDP model may surpass 90% due to the majority of the healthy cases included in the training and testing data. In such cases, it is extremely significant to measure the model performance by additional metrics such as precision, recall, confusion matrix, actual-predicted diagrams/values, mean absolute error rates, etc. This may constitute an additional challenge since it is necessary to be aware of such situations. In addition, it may be compelling to select suitable performance metrics for the model evaluation.
• Interdisciplinary collaboration: Machine learning for rotating machinery is a multi-disciplinary research area. Hence, expertise from disciplines such as mechanical and computer sciences may be required. Cooperation among the corresponding experts is likely needed so as to accomplish machine learning implementations.
• Real-time adaptation: A fundamental challenge is developing an effective intelligent model that is adaptable for real-time applications because machinery components are significantly affected by environmental factors such as temperature, pressure, humidity, pollutants, noise, etc. In addition, machines usually do not operate as they are assumed to do in controlled real or virtual environments such as laboratories or simulations. Hence, it is essential to develop a model that is resilient against such factors. On the other hand, it is also challenging to assess the performance of the IFDP model in real-world settings due to time consumption and potential failures that may result in unnecessary costs.
• Domain adaptation: In general, intelligent techniques are trained and tested considering a single benchmark or commercial dataset. Although pursuing such an approach is usually fruitful, it may still give fallacious results regarding the performance of the developed model when it is tested with unseen data acquired from the same kind of component from a different machine. Therefore, it is necessary to constitute an intelligent method that is able to give accurate predictions regarding the same domain (component) of different machines.
• Fault types and compound faults: Another significant challenge is to determine the limits in terms of fault type or fault occurrences when developing an IFDP model. It is necessary to consider all kinds of faults that are likely to occur during an operation. In several cases, a fault development may cause to arise another fault in the same or different component, which may adversely impact the model performance. Hence, it is necessary to pursue a deterministic approach and consider compound faults when constituting an IFDP approach for rotating machines.

## 8 Research directions

Based on the studies in the literature and recent challenges, the following research directions may be considered in further studies (RQ11).

1. Real-world dataset: The majority of the dataset is fabricated in laboratory conditions where environmental or other disruptive factors are discarded. Most of the studies related to the IFDP of rotating machines used such benchmark datasets to assess their proposed approach. On the other hand, it is necessary to consider these factors because they somehow affect the indicators of a machine. Hence, future studies may comprise or propose datasets in real-world settings for intelligent fault analysis of rotating machines although collecting such data may take a significant amount of time. By doing so, it may be possible to constitute IFDP models that are able to handle the changes in environmental and operational parameters.
2. Component and fault-specific models: Although it is possible to constitute a generalizable model regarding the fault analysis of multiple components, it may be more beneficial to develop an IFDP model for each type of component and tune its parameters considering the type of fault. By doing so, the complexity of the problem would be decreased since only the faults of a specific component are considered instead of taking numerous kinds of faults belonging to various components. Hence, further studies may investigate the effectiveness of a component and fault-specific IFDP approach.
3. Multiple or Compound faults: Rotating machines may include multiple faults occurring in the same or different components. There are scarce studies in the literature that measures the effectiveness of the IFDP approaches regarding the compound fault condition in rotating machines. Future studies may measure the effectiveness of IFDP approaches considering compound faults as such situations may be observed in real-world settings.
4. Complete Fault Analysis: Most studies proposed IFDP approaches by considering fault detection, fault identification, fault localization, or fault prognosis procedures. Developing effective intelligent techniques that are capable to conduct a complete fault analysis where all procedures are covered will significantly contribute to the related community. For real-world settings, it is also suggested to combine different ML methods in a fault analysis pipeline where each algorithm is responsible to handle a specific task. For instance, one semi-supervised model may be developed for fault detection while one supervised model may in charge to distinguish the environmental anomalies that affect to data used for fault detection. By combining ML approaches in such a way, it may be possible to avoid deceiving results and constitute a more robust IFDP method.
5. Ensemble Methods: The majority of IFDP techniques developed for rotating machines considered deep learning (DL) approaches due to the positive aspects regarding automated feature extraction and high accuracy. On the other hand, these approaches may require expensive powerful devices, which may not be suitable for embedded systems. In addition, DL methods are generally time-consuming, which is not practical for real-world applications. Hence, the ensemble learning techniques are needed to be examined in more depth since they may not demand a high computational load and, at the same time, are accurate and robust.
6. Semi-supervised and Unsupervised Methods: The majority of the studies take supervised approaches into account where labeled instances are used to train the IFDP model. On the other hand, using supervised methods may not be practical in real-world settings since it is extremely challenging to constitute a dataset in which all possible faults that may occur in a component are included. This would take so much time, and effort and may result in undesirable consequences including the complete failure of a machine. Practically, semi-supervised and unsupervised approaches are more convenient for fault detection of rotating machines. In addition, having numerous and a wide variety of labeled data may not be handled by a proposed approach for fault diagnosis. In such cases, employing a semi-supervised or unsupervised approach may be more fruitful for fault detection instead of trying to identify and localize the fault.
7. Human-in-the-Loop: As a recent popular approach, human-in-the-loop aims to combine supervised learning with human feedback active learning where an intelligent approach makes more accurate decisions as it receives correct commands or inputs from a human. Future studies related to the IFDP of rotating machines would significantly benefit from such an approach, especially in fault diagnosis procedures where the fault is identified and localized. By employing human-in-the-loop it is possible to constitute more reliable IFDP approaches with a smaller size of initial data since the method will reinforce its learning by receiving a command that includes the details of the condition of the machine.
8. Data Augmentation: The acquisition of the healthy data of a rotating machine component is easy because the machine generally operates in its healthy condition. On the other hand, gathering faulty data from an industrial machine is a challenging process since the machine may completely fail or even be destroyed during the data collection procedure. This raises another challenge, especially in the fault detection procedure regarding the amount of healthy and faulty data. Although there are numerous studies that examined data augmentation to obtain higher amounts of data, it is still worth investigating as handling and the requirement of the data may become compelling, especially in more complex rotating machines.
9. Model Assessment: Interpreting a performance of a developed intelligent model based on only accuracy may give fallacious results, especially for unbalanced data. An IFDP model may give high accuracy and still make a false prediction. For instance, consider a dataset with 1000 instances, which is split by 900 in the favor of “healthy condition” and 100 for “faulty condition”. A prediction accuracy of 90% may be erroneous since all accurately predicted instances may cover the “healthy state” (900 of 1000) whereas the entire “faulty state” instances are mispredicted as healthy. Hence, it is necessary to include other metrics such as precision and recall. Regarding fault-specific tasks, the confusion matrix is another required metric to assess the model performance in a fault-specific domain. For regression-based approaches, using solely coefficient of determination (R2), root mean squared error, or mean absolute error may present misleading conclusions regarding the model performance. In addition to such metrics, it is critical to explore the actual and predicted values and interpret how close they are. Hence, the future classification or regression-based IFDP approaches may be assessed in more detail and comprehensively by including additional metrics. In addition to performance metrics, it is strongly recommended to evaluate an IFDP model with unseen data and/or different benchmark or real-world datasets.

## 9 Conclusions

This study presents a comprehensive review of IFDP procedures for rotating machinery by minding all the existing challenges. The proposed IFDP approaches are widely discussed considering the fault analysis strategies, deemed data sources, data types, data fusion techniques, machine learning techniques within the frame of the fault type, and compound faults that occur in components such as bearings, gear, rotor, stator, shaft, and other parts. Besides, the existing challenges in developing, evaluating, and using IFDP approaches are presented. Finally, future directions are presented considering the issues and gaps existing in the literature to the community to propose effective IFDP approaches for rotating machines.

## Author contribution statement

All authors listed have significantly contributed to the development and the writing of this article.

## Data availability statement

No data was used for the research described in the article.

## Declaration of competing interest

The authors declare that they have no known competing financial interests or personal relationships that could have appeared to influence the work reported in this paper.
